# The SUMO-Conjugase Ubc9 Prevents the Degradation of the Dopamine Transporter, Enhancing Its Cell Surface Level and Dopamine Uptake

**DOI:** 10.3389/fncel.2019.00035

**Published:** 2019-02-08

**Authors:** Etienne Cartier, Jennie Garcia-Olivares, Eric Janezic, Juan Viana, Michael Moore, Min Landon Lin, Jeffrey L. Caplan, Gonzalo Torres, Yong-Hwan Kim

**Affiliations:** ^1^Department of Biological Sciences, Delaware State University, Dover, DE, United States; ^2^National Institute of Mental Health (NIMH), Bethesda, MD, United States; ^3^Imaging Core, Delaware State University, Dover, DE, United States; ^4^Department of Neuroscience and Department of Pharmacology, University of Florida, Gainesville, FL, United States; ^5^BioImaging Center, University of Delaware, Newark, DE, United States

**Keywords:** dopamine transporter, SUMOylation, Ubc9, ubiquitin, lysosome, proteostasis

## Abstract

The dopamine transporter (DAT) is a plasma membrane protein responsible for the uptake of released dopamine back to the presynaptic terminal and ending dopamine neurotransmission. The DAT is the molecular target for cocaine and amphetamine as well as a number of pathological conditions including autism spectrum disorders, attention-deficit hyperactivity disorder (ADHD), dopamine transporter deficiency syndrome (DTDS), and Parkinson’s disease. The DAT uptake capacity is dependent on its level in the plasma membrane. *In vitro* studies show that DAT functional expression is regulated by a balance of endocytosis, recycling, and lysosomal degradation. However, recent reports suggest that DAT regulation by endocytosis in neurons is less significant than previously reported. Therefore, additional mechanisms appear to determine DAT steady-state level and functional expression in the neuronal plasma membrane. Here, we hypothesize that the ubiquitin-like protein small ubiquitin-like modifier 1 (SUMO1) increases the DAT steady-state level in the plasma membrane. In confocal microscopy, fluorescent resonance energy transfer (FRET), and Western blot analyses, we demonstrate that DAT is associated with SUMO1 in the rat dopaminergic N27 and DAT overexpressing Human Embryonic Kidney cells (HEK)-293 cells. The overexpression of SUMO1 and the Ubc9 SUMO-conjugase induces DAT SUMOylation, reduces DAT ubiquitination and degradation, enhancing DAT steady-state level. In addition, the Ubc9 knock-down by interference RNA (RNAi) increases DAT degradation and reduces DAT steady-state level. Remarkably, the Ubc9-mediated SUMOylation increases the expression of DAT in the plasma membrane and dopamine uptake capacity. Our results strongly suggest that SUMOylation is a novel mechanism that plays a central role in regulating DAT proteostasis, dopamine uptake, and dopamine signaling in neurons. For that reason, the SUMO pathway including SUMO1, SUMO2, Ubc9, and DAT SUMOylation, can be critical therapeutic targets in regulating DAT stability and dopamine clearance in health and pathological states.

## Introduction

The dopamine transporter (DAT) is a plasma membrane-spanning protein responsible for the termination of dopaminergic neurotransmission *via* reuptake of released dopamine from the presynaptic terminals in the central nervous system, which is the main mechanism for terminating dopamine transmission in the brain (Hong and Amara, 2013; Rudnick et al., 2014; German et al., 2015). The DAT is the molecular target for commonly abused drugs including cocaine, amphetamine, and methamphetamine (Hong and Amara, 2013; Rudnick et al., 2014; German et al., 2015). In addition, several coding variants have been described in autism spectrum disorders, attention-deficit hyperactivity disorder (ADHD), dopamine transporter deficiency syndrome (DTDS), and Parkinson’s disease (Kurian et al., 2009, 2011; Sakrikar et al., 2012; Bowton et al., 2014; Hansen et al., 2014; Ng et al., 2014; Cartier et al., 2015; Herborg et al., 2018). Therefore, understanding the molecular mechanisms of DAT availability and functional expression is crucial to identify the regulatory basis of dopamine signaling in health and disease.

The dopamine uptake capacity by DAT is dependent on its availability in the plasma membrane (Sakrikar et al., 2012; Hong and Amara, 2013; Bowton et al., 2014; Rudnick et al., 2014; Cartier et al., 2015; German et al., 2015). Although defective proteostasis has been demonstrated to be a cause of many neurodegenerative diseases, the degradation mechanisms regulating DAT availability are not well understood. Recent studies have linked mutations in the human DAT gene causing DTDS, to be responsible for DAT misfolding, degradation, and reduced level in the plasma membrane (Kurian et al., 2009, 2011; Hetz and Mollereau, 2014; Ng et al., 2014; Ciechanover and Kwon, 2015; Beerepoot et al., 2016; Kasture et al., 2016). Remarkably, the use of pharmacological chaperones *in vitro* enables to rescue some of the DAT misfolded mutants (Beerepoot et al., 2016; Kasture et al., 2016). Thus, comprehending the regulatory mechanisms that modulate DAT folding and degradation may have a profound therapeutic impact.

Studies have demonstrated that DAT functional expression is regulated by a balance of endocytosis, recycling, and lysosomal degradation (Daniels and Amara, 1999; Loder and Melikian, 2003; Miranda et al., 2005, 2007; Sorkina et al., 2005; Hong and Amara, 2013; Wu et al., 2015). In heterologous systems, DAT targeting for lysosomal degradation is activated by protein kinase C (PKC) and requires its modification by poly-ubiquitin (Daniels and Amara, 1999; Loder and Melikian, 2003; Miranda et al., 2005, 2007; Sorkina et al., 2005; Hong and Amara, 2013). However, a few reports have shown that DAT stability is mediated by the proteasome (Bjerggaard et al., 2004; Jiang et al., 2004). Recent studies have demonstrated that DAT endocytosis in brain slices is active only in a fraction of total DAT in the plasma membrane. Moreover, additional reports in neurons have questioned that activation of PKC induces DAT internalization and lysosomal degradation, in contrast to results obtained in heterologous systems (Eriksen et al., 2009; Gabriel et al., 2013; Block et al., 2015). Therefore, further mechanisms in neurons appear to determine the DAT degradation and functional expression in the plasma membrane. This supports the possibility that neuronal DAT availability is not only regulated *via* internalization but also by degradative pathways that target DAT through post-translational modifications. Since DAT can be a target for a variety of post-translational modifications including ubiquitination, palmitoylation, glycosylation, and phosphorylation (Li et al., 2004; Miranda et al., 2005, 2007; Foster and Vaughan, 2011; Bermingham and Blakely, 2016), it is possible that additional post-translational modifications may regulate DAT availability. Recent studies have established the modification by the Small Ubiquitin-like Modifier (SUMO) as a prominent signal in the central nervous system, which is an important regulator of protein stability (Dorval and Fraser, 2007; Flotho and Melchior, 2013; Henley et al., 2014). There are two main SUMO paralogs reported in humans, SUMO1 and SUMO2/3. SUMO1 shares ~50% identity with SUMO2, while SUMO2 and 3 differ in only three NH2-terminal residues and cannot be discriminated by immunoblotting (Dorval and Fraser, 2007; Flotho and Melchior, 2013; Henley et al., 2014). SUMO can associate with its targets by protein-protein interaction or direct conjugation. SUMOylation involves the reversible modification of SUMO to lysine residues on the target protein (Dorval and Fraser, 2007; Flotho and Melchior, 2013; Henley et al., 2014). The SUMOylation pathway resembles that of ubiquitination, as SUMO is activated by an E1 dimer complex, the SUMO-activating enzyme 1 and 2 (SAE1/SAE2) in humans. Then SUMO is transferred to the single E2 conjugating enzyme, Ubc9. SUMOylation can proceed with the presence of Ubc9 alone, however, Ubc9 normally associates with specific E3 SUMO ligases (Hayashi et al., 2002; Dorval and Fraser, 2007; Lee et al., 2011; Flotho and Melchior, 2013; Henley et al., 2014).

Ubc9 is mostly expressed in the nucleus and cytoplasm and has been mainly characterized as an enzyme necessary for a variety of cellular processes, including nuclear transport, transcriptional regulation, and apoptosis (Hayashi et al., 2002; Dorval and Fraser, 2007; Lee et al., 2011; Flotho and Melchior, 2013; Henley et al., 2014). However, accumulating evidence supports the extranuclear roles of Ubc9 and its presence in the plasma membrane (Giorgino et al., 2000; Rajan et al., 2005; Tang et al., 2005; Liu et al., 2007; Martin et al., 2007; Kruse et al., 2009; Dustrude et al., 2013; Sun et al., 2014; Benson et al., 2017). In excitable cells, the covalent and non-covalent associations of SUMO have been demonstrated in several proteins including the TRPM4, K2P1, and Kv1.5 ion channels, as well as metabotropic glutamate receptors, neuronal kainate receptors, and PKC (Giorgino et al., 2000; Rajan et al., 2005; Tang et al., 2005; Liu et al., 2007; Martin et al., 2007; Kruse et al., 2009; Dustrude et al., 2013; Sun et al., 2014; Benson et al., 2017). In addition, SUMO can be conjugated to cytoplasmic proteins including α-synuclein whose aggregation induces Lewy body formation in Parkinson’s disease pathology (Krumova et al., 2011; Eckermann, 2013; Rott et al., 2017; Rousseaux et al., 2018). α-synuclein SUMOylation may increase its toxic accumulation by reciprocally down-regulating α-synuclein ubiquitination (Krumova et al., 2011; Eckermann, 2013; Rott et al., 2017; Rousseaux et al., 2018). In the same way, Huntingtin can also be SUMOylated, by which its degradation can be impaired by decreasing its ubiquitination, enhancing Huntingtin capacity to build toxic aggregates (Steffan et al., 2004; O’Rourke et al., 2013). Therefore, for some pathologically significant proteins, the crosstalk between the SUMO and ubiquitin systems determines their steady-state level and aggregation state (Steffan et al., 2004; Tatham et al., 2008; O’Rourke et al., 2013; Liebelt and Vertegaal, 2016; Rott et al., 2017; Rousseaux et al., 2018).

Here, we demonstrate that DAT is associated with SUMO1 in dopaminergic N27 and human embryonic kidney cells (HEK)-DAT 293 cells. Overexpressed SUMO1 and Ubc9 activities enhance DAT SUMOylation, which leads to reduced DAT ubiquitination and degradation, increasing DAT steady-state level. The Ubc9 and SUMO enhance DAT stability, which is crucial to determine the increase of dopamine uptake capacity in the plasma membrane. Our results strongly suggest that SUMOylation is a novel mechanism that plays an important role in regulating dopamine clearance and signaling in neurons.

## Materials and Methods

### Cell Lines and Culture

N27 parental cells were obtained from EMD Millipore and correspond to a rat dopaminergic cell line harvested from E12 rat mesencephalic tissue and transfected with SV40 to immortalize the cell line that expresses DAT and tyrosine hydroxylase (TH; Adams et al., 1996; Clarkson et al., 1998; Lazzara et al., 2015). N27 enhanced green fluorescent protein (EGFP), Ubc9-EGFP, and DAT-EYFP stable cell lines were developed by transfecting plasmids to parental N27 dopaminergic cells, using neomycin resistant plasmids (based on pEGFP or pEYFP vectors, Takara Clontech). N27 cells were cultured in the cell culture media (10% FBS in RPMI 1640, Invitrogen, Carlsbad, CA, USA) with 1% Penicillin-Streptomycin and transfected using Lipofectamine 2000 (Invitrogen), according to the instructions of the manufacturer. Green fluorescence was visualized under a fluorescent microscope, 48 h after transfection. Geneticin (Gibco) was added as a selection marker at the concentration of 500–1,000 μg/ml for about 2 weeks. Fluorescent positive green (EGFP) or yellow cells (EYFP) were plated in multi-well selection plates. Clusters of green or yellow fluorescent positive cells were isolated and plated in 60 mm dishes. Geneticin concentration was maintained at 500 μg/ml for another 2 weeks and then lowered to 200 μg/ml. HEK-293 cells with stable expression of DAT (HEK-DAT) were cultured in 200 μg/ml geneticin. Parental HEK and HEK-DAT cells were cultured in Dulbecco’s modified Eagle’s medium supplemented with 10% FBS plus Penicillin-Streptomycin 100 U/ml–100 μg/ml (Sigma) at 37°C in a 5% CO_2_ incubator.

### Plasmids and Transfections

The EGFP-N1 vector was obtained from Clontech. SUMO1-HA, SUMO2-HA, and SUMO1-ECFP cDNAs were gifts from Ronald T. Hay (University of Dundee). YFP-CFP tandem (Cerulean-Venus, C5V), YFP alone (Venus), and CFP alone (Cerulean) vectors were obtained from Addgene, which were deposited by Steven Vogel (Koushik et al., 2006). hDAT-ECFP and hDAT-EYFP were obtained from Alexander Sorkin (Sorkina et al., 2003). Ubc9-EGFP was a gift from Jo Morris (King’s College London) and Ubc9-HA and Ubc9-HA C93S (inactive/dominant negative, Ubc9CS) were obtained from Mary Dasso at NIH. hDAT-HA was used as reported by Torres et al. (2003). FLAG-tagged Ubiquitin cDNA was obtained from Ivan Dikic at Goethe University. As a control, empty vector pcDNA 3.1 from Invitrogen was used. Transfections with cDNA proceeded on 60 mm cell seeded dishes with 4–5 μg of total cDNA and Lipofectamine 2000 on a 1:1 ratio to avoid cell toxicity. Cells were assayed for different experiments, 48 h after transfection. Fugene (Promega) was also used for HEK-DAT transfections using the manufacturer’s indications.

### Immunocytochemistry

#### Immunostaining of N27 Cells

N27 cells were plated at low confluence on glass coverslips. Cells were washed three times with cold phosphate buffered saline (PBS) without Ca^2+^ or Mg^2+^ (137 mM NaCl, 2.7 mM KCl, 4.3 mM Na_2_HPO_4_, and 1.47 mM KH_2_PO_4_) on ice, fixed with 4% paraformaldehyde (PFA) for 10 min and washed with PBS at room temperature. Fixed cells were permeabilized with 0.1% Triton X-100 and 0.5% bovine serum albumin (BSA) for 3 min, blocked with 5% BSA for several hours at room temperature, and incubated with anti-DAT primary antibodies (rabbit “EL2,” 1:1,000, EMD-Millipore: AB5802 or rat “MAB,” 1:1,000, EMD-Millipore MAB369) or rabbit anti-TH primary antibody (1:1,000, EMD-Millipore AB152) in 1% BSA/PBS at 4°C overnight. Then cells were extensively washed three times in 1% BSA/PBS and incubated with secondary goat anti-rabbit Alexa 594 (1:4,000, ThermoFisher Scientific) or goat anti-rabbit Alexa 488 (1:4,000, ThermoFisher Scientific) for 1 h. Cells were extensively washed three more times in 1% BSA/PBS and then incubated with DAPI in PBS for 10 min, followed by a brief washing in ultra-pure water. Coverslips were mounted on glass using ProLong Diamond antifade mounting medium (Thermo). Images were obtained using a Zeiss 780 Multiphoton Confocal microscope with separate laser lines for 405 nm, 488 nm, 514 nm, and 561 nm to excite DAPI, Alexa 488/GFP, YFP, and Alexa 594, respectively. All images were acquired with a Plan-Apochromat 40×/1.4NA Oil DIC objective (Zeiss). Images were processed using Zeiss ZEN software and sampled at optimal pixel density (PD) in the X and Y with a line average of 4, and a zoom factor of 1.2. Z-stacks were set up with optimal z sectioning for optimal overlap and processed into maximum intensity projections.

#### Immunostaining of HEK-DAT Cells

HEK-DAT cells on coverslips were transfected using Fugene with the Ubc9-HA construct and fixed with cold 4% PFA for 15 min after 48 h of the transfection, and permeabilized with 0.5% Triton X-100 for 5 min. Fixed cells were washed in PBS and blocked with 1% BSA for 1 h and incubated with two primary antibodies overnight: rat anti-DAT (EMD-Millipore, MAB 369) and mouse anti-HA (Sigma, clone HA-7). Cells were then washed several times in PBS + 0.01% Tween 20 (PBS-T), incubated with secondary goat antibody conjugated to Alexa 488 (green) or Alexa 546 (red; 1:4,000 dilution; Thermo). Cells were washed three times in PBS-T and then incubated with DAPI in PBS solution for 10 min, followed by washing and mounting on glass. Nikon A1 laser-scanning confocal microscope (Nikon Corporation, Tokyo, Japan) was used for collecting images. Green fluorophore was excited using a 488 nm laser (Argon laser), blue fluorophore (DAPI) at 405 nm, and red fluorophore was excited at 561 nm (Sapphire laser).

### Surface Biotinylation in HEK-DAT and N27 Cells

HEK-DAT and N27 cells were seeded on 60 mm dishes. HEK-DAT cells were transfected with plasmid cDNA using Fugene (Promega) or Lipofectamine 2000 (Invitrogen) as the manufacturer’s instructions. HEK-DAT cells were biotinylated with 1.5 mg/ml EZ-Link biotin (Thermo) in cold PBS at 4°C for 1 h, 72 h after transfection. N27 cells were incubated with 6 μM 2-D08 (EMD Millipore) or vehicle for 48 h and biotinylated with 2.5 mg/ml EZ-Link biotin in cold PBS at 4°C for 1 h. To improve DAT recovery in N27 cells, two equally seeded 60 mm dishes were used for each condition. In all cases, biotin excess was quenched by two washes in 0.1 M glycine**/**PBS, and cells were solubilized using RIPA buffer (50 mM Tris-HCl, 150 mM NaCl, 1 mM EDTA, 1% Triton X-100, 0.1% sodium dodecyl sulfate, and 0.5% sodium deoxycholate). Protein concentration for each assay was immediately quantified by the BCA protein assay (Thermo). An equal amount of protein was incubated with Neutravidin beads (Thermo) overnight, followed by washing three times in cold RIPA. Biotinylated membrane proteins were eluted in 50 μl of Laemmli sample buffer. Eluted proteins were loaded and separated by SDS-PAGE and DAT was detected by immunoblotting using rat anti-DAT antibody (MAB, EMD Millipore) in both HEK-DAT and N27 cells. “Total Protein” (input) samples were taken from the biotinylated cell extracts before the use of beads. An equal amount of protein was loaded for every experiment and repeated several times.

### [^3^H]-Methyl-4-Phenylpyridinium (MPP^+^) Uptake Assay

N27 cells were plated in a 24-well plate pre-coated with poly-D-Lysine. Cells were incubated with [^3^H]-methyl-4-phenylpyridinium (MPP^+^) at 37°C for 15 min. For uptake experiments, cells were incubated with specific DAT inhibitors: GBR12935 (10 μM) and mazindol (100 μM), NET inhibitor: desipramine (10 μM), and SERT inhibitor: citalopram (10 μM). All monoamine inhibitors were preincubated for 10 min before the addition of 50 nM of [^3^H]-MPP^+^ (79.8 Ci/mmol, Perkin Elmer) in RPMI cell culture media, and were maintained during the uptake. The uptake of [^3^H]-MPP^+^ was blocked after 15 min in ice-cold NaCl-free uptake buffer. Cells were gently washed twice and lysed in 0.4 ml of 1% SDS. The accumulated [^3^H]-MPP^+^ was measured by a liquid scintillation counter (Beckman).

### FRET Studies

The quantification of fluorescent resonance energy transfer (FRET) between DAT-EYFP and SUMO1-ECFP was performed by the acceptor photobleaching method (Karpova et al., 2003; Karpova and McNally, 2006). Our protocol closely followed the methodology and controls determined by Karpova et al. (2003) and Karpova and McNally (2006). Stably expressing DAT-EYFP or parental N27 cells were transfected with different constructs to produce the necessary positive and negative controls for the DAT-YFP/SUMO1-CFP pair. Used constructs: (1) YFP-CFP tandem (C5V) as specified (Koushik et al., 2006); (2) CFP alone (unconjugated); (3) CFP+YFP (both unconjugated); (4) DAT-CFP as specified by Sorkina et al. (2003); (5) YFP alone (unconjugated). All constructs were transfected into N27 parental cells or N27 DAT-YFP cells, depending on the experiment. N27 cells were fixed with 4% PFA at room temperature for 30 min, washed in PBS, and mounted on glass. Spectral FRET proceeded with a Zeiss 880 confocal microscope equipped with an ultra-sensitive GaAsP detector that allows visualization of weak fluorescent signals with lower photobleaching. The Zeiss EC Plan-Neofluar 40×/1.30NA oil objective was used for all FRET studies. The image was acquired with a zoom factor of 3 in a cropped image dimension of 1,024 × 256 allowing for faster acquisition times. The light path was set up with a 458/515 main beam splitter and imaged with a 458 nm laser. The entire emission spectra were collected within the band window (410–695 nm) using the GaAsP detector. The Argon laser 514 nm line was used to photobleach YFP-tagged expressing protein (i.e., DAT-YFP). Time series Lambda stacks were acquired with 3 pre-bleach images and 10 post-bleach images. Background fluorescence was subtracted from all images. YFP photobleaching proceeded at 514 nm with 50% laser power. The nuclei were always excluded from selected regions and DAT-YFP expressed in membranes (plasma and endoplasmic reticulum) was focused. Spectral values obtained from pre- and post-photobleaching were analyzed using Zeiss ZEN Software. FRET values in Figure 2C were calculated from peak fluorescence intensities for each region of interest as shown in Figure 2B. FRET efficiencies shown in Figure 2D and Table 2 were calculated based on the protocols and indications determined by Karpova et al. (2003) and Karpova and McNally (2006). FRET images are presented in pseudo-color mode (ZEN-Rainbow2 pseudo-color, where low intensities are depicted in violet/blue, medium intensities in yellow, and high intensities in red).

**Figure 1:**
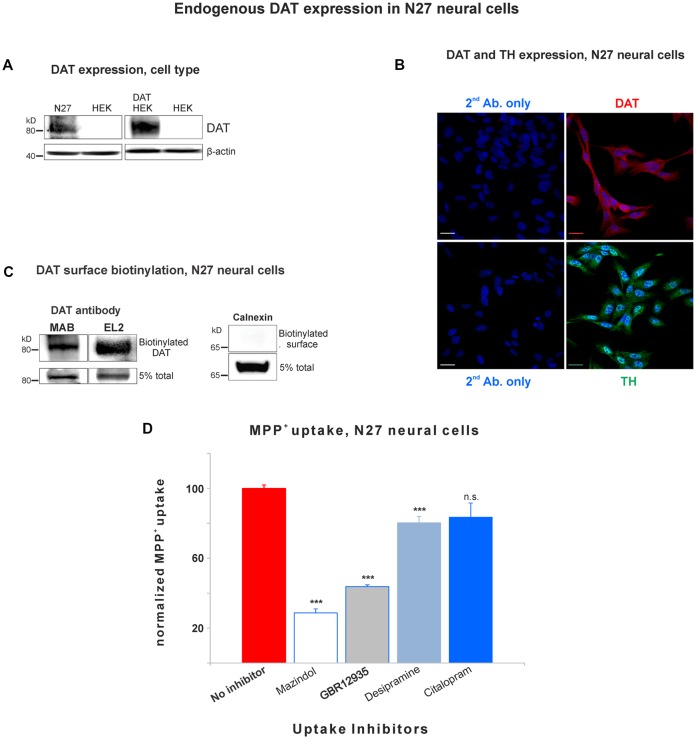
Dopamine transporter (DAT) is endogenously expressed in the plasma membrane of N27 cells. **(A)** Rat dopaminergic N27 cells express DAT endogenously, while human embryonic kidney cells (HEK)-293 cells do not express DAT unless DAT was exogenously introduced. **(B)** Representative confocal microscopy images display specific staining of DAT and tyrosine hydroxylase (TH) in N27 cells. The specificity of DAT rabbit antibody (EL2) was compared with secondary antibody only with DAPI nuclear labeling as a negative control. TH staining indicates that N27 cells are dopaminergic. Bar size: 20 μm. **(C)** DAT is detected in the plasma membrane fraction. After isolating biotinylated cell surface proteins, both MAB and EL2 antibodies specifically recognize the surface DAT. Calnexin, a marker for ER, was included as a negative control. **(D)** The methyl-4-phenylpyridinium (MPP^+^) was predominantly taken up by DAT in N27 cells, while the effect by NET and SERT was marginal or not significant. The [^3^H]-MPP^+^ uptake was measured with four different transporter blockers: mazindol (NET and DAT inhibitor), GBR12935 (DAT inhibitor), desipramine (NET inhibitor), and citalopram (SERT inhibitor). Values represent mean ± SE and statistical significance from control (***p < 0.001) was determined by one-Way analyses of variance (ANOVA) with Tukey’s range test. Data represent the values of three to five independent experiments. ns, not significant.

**Figure 2:**
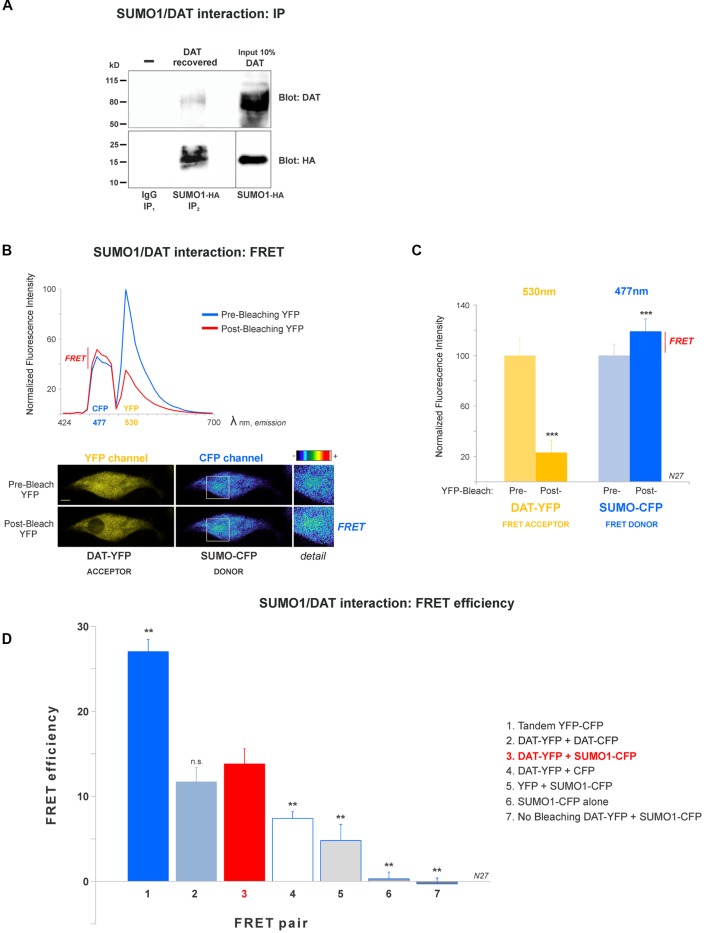
DAT is physically associated with Small Ubiquitin-like Modifier 1 (SUMO1) in N27 cells. **(A)** DAT co-precipitates with SUMO1-HA in HEK-DAT cells. SUMO1 was immunoprecipitated with anti-SUMO1 or IgG (rabbit) antibodies and immunoblotted with the anti-HA antibody. Co-immunoprecipitated DAT was detected with anti-DAT (MAB) antibody. **(B)** The spectral fluorescent resonance energy transfer (FRET) histogram of DAT-YFP + SUMO1-CFP suggests their association. The photobleaching of DAT-YFP (FRET acceptor, peak fluorescence at 531 nm) increases fluorescence of SUMO1-CFP (FRET donor, peak fluorescence at 477 nm). Confocal images are shown at the bottom, featuring DAT-YFP fluorescence before and after photobleaching and the increase of SUMO1-CFP fluorescence shown in pseudocolor intensity levels. **(C)** Quantification of peak fluorescence values, as shown in **(B)**, from FRET donor before and after photobleaching suggests the physical association of DAT-YFP and SUMO1-CFP. DAT-YFP acceptor and SUMO1-CFP donor peak fluorescence are shown before and after acceptor (DAT-YFP) photobleaching (three independent experiments). All values were normalized as average ± SE and statistical significance (***p < 0.001) was determined by a two-sided, Student’s *t*-test. **(D)** The FRET efficiency of the DAT-YFP + SUMO1-CFP pair was significantly higher than those of negative control groups. The FRET efficiencies for positive and negative controls are displayed. All values were normalized as average ± SE and statistical significance (**p < 0.01) was determined by one-way ANOVA with a Tukey’s *post hoc* test (three independent experiments). ns, not significant.

**Table 1:** The concentrations of monoamine transporter inhibitors used in the MPP^+^ uptake and their IC50s for dopamine transporter (DAT), NET, and SERT are displayed.

Inhibitor used concentration and monoamine transporters IC50				
Inhibitor	Concentration used	DAT (IC50 nM)	NET (IC50 nM)	SERT (IC50 nM)
Mazindol*	100 μM	28.8	3.3	160
GBR12935*	10 μM	22.4	235	6,800
Desipramine*	10 μM	82,000	4.2	64
Citalopram^#^	10 μM	41,000	8,800	1.8

Ref: *Eshleman et al. (1999), ^#^Hyttel (1982).

**Table 2:** Values for fluorescent resonance energy transfer (FRET) efficiency for positive and negative controls (*n* = 11–21) for three independent experiments on each case.

FRET efficiencies values and statistical significance		
FRET Pair	FRET efficiency	Significance
DAT-YFP + SUMO1-CFP	13.8 ± 1 0.8	-
YFP-CFP tandem (C5V)	27.2 ± 1 0.4	**
DAT-YFP + DAT-CFP	11.7 ± 1 0.7	non sig.
DAT-YFP + CFP	7.4 ± 0.8	**
YFP + SUMO1-CFP	4.8 ± 1 0.9	**
YFP + CFP	8.6 ± 1 0.2	*
SUMO1-CFP	0.3 ± 0.8	**
DAT-YFP + SUMO1-CFP (No.B)	−0.3 ± 0.7	**

All values were normalized as average ± SE and statistical significance (**p < 0.01, *p < 0.05) was determined by one-way ANOVA with a Tukey’s *post hoc* test.

### Cycloheximide Protein Chase

The protein chase study was performed based on Daniels and Amara with modifications (Daniels and Amara, 1999). Ubc9-GFP, GFP transfected or N27 parental cells were equally plated on 60 mm dishes and incubated with 100 μg/ml cycloheximide for 1 h in the culture media when time point 0 (*T* = 0 h) was obtained. Further incubations with 100 μg/ml cycloheximide were performed until the experiment was terminated at different time points (*T* = 2 h, 6 h, 12 h, 18 h, or 24 h). Cell extracts were obtained in 200 μl of 1× Laemmli sample buffer, tip sonicated briefly for solubilization (1–2 s, and centrifuged at 16,000 *g* for 10 min for collecting insoluble debris. Drugs or vehicle were added to the media at *T* = 0 h for the rest of the chase study, which was terminated as described above. Cell extracts were separated by 4%–20% SDS-PAGE gels and DAT was detected by immunoblotting with rabbit anti-DAT antibody (EL2, Millipore). Equal loading was determined by stripping and re-probing against β-actin.

### Quantitative mRNA Measurement

A quantitative real-time PCR (qRT-PCR) was performed to determine the level of DAT mRNA, with β-actin as a housekeeping gene. An equal number of cells for each cell line were collected for RNA extraction using the TRIzol^®^ Reagent (Life Technologies). Briefly, cells were homogenized in TRIzol and incubated at room temperature for 5 min. Following homogenization, chloroform was added and the reaction was incubated at room temperature for 3 min. The samples were then centrifuged at 12,000 *g* at 4°C for 15 min. One-hundred percent isopropyl alcohol was added to the upper aqueous layer (containing the RNA), and incubated at room temperature for 10 min, followed by 10 min of centrifuging at 4°C. Then, the supernatant was removed and the RNA pellet was washed with 75% ethanol and centrifuged at 7,500 *g* at 4°C for 5 min. After the supernatant was discarded, the RNA was suspended in DEPC-treated water. Immediately following the extraction of RNA, 5 μg of total RNA for each cell line was used to produce cDNA using the SuperScript^®^ IV First-Strand Synthesis kit (Invitrogen). The RNA was added to 0.5 μl of random hexamers (50 ng/μl), 0.5 μl of 50 μM Oligo d(T)20, and DEPC-treated water and incubated at 65°C for 5 min. Following the initial incubation, the RNA/primer mixture was added to a mixture of 5× SSIV buffer (4 μl), 100 nM DTT (1 μl), RNAseOUT™ Recombinant RNase Inhibitor, and 1 μl of SuperScript^®^ IV Reverse Transcriptase (200 units/μl). The reaction was incubated at room temperature for 10 min, and then at 55°C for 20 min. Following incubation, the reaction was brought to 80°C for 10 min to prevent further synthesis. For the qRT-PCR, we used the protocol of SYBR-Green I Master kit using LightCycler^®^ 480 system (Roche). The reaction was run in a 384 well plate with 10 μl per reaction well. Each well consisted of 1.5 μl H_2_O, 5 μl 2× Master Mix, 0.5 μl each of the forward and reverse primers (for a final concentration of 5 nM each), and 2.5 μl of the cDNA as described above (total of 20 ng). The plate was then centrifuged at 1,500 *g* for 1 min. The parameters for the reactions were: 95°C for 10 min, followed by 50 cycles of 95°C for 30 s, 60°C for 10 s, and 72°C for 10 s. The fluorescence was recorded during the 72°C step in order to determine the Crossing point (C_p_) value. The primers were chosen based on Primer3Plus Software. The primers are: DAT-forward 5′-AAAAAGGTAGCGGGGTCAGT-3′; DAT-reverse 5′-GAAGGCAGGTGCAAGAGTTC-3′; β-actin-forward 5′-AGCCATGTACGTAGCCATCC-3′; β-actin-reverse 5′-CTCTCAGCTGTGGTGGTGAA-3′. Each trial was in triplicate, and three trials were collected for a total of nine independent samples for each gene.

### Ubc9 Interference RNA (RNAi)

Interference RNA (RNAi) cocktail was prepared by a combination of two selected RNAi constructs from IDT (Coralville, IA, USA; labeled as RNAi-1: RNC.RNAI.N013050.12.1 and RNAi-2: RNC.RNAI.N013050.12.3) pre-designed for rat Ubc9 mRNA inhibition. As a negative control, RNAi sequence designed by IDT was used (Negative Control 1: NC1). The RNAi constructs were transfected as the manufacturer indicated. In short, 80 pmoles of total RNAi (1×) was incubated in Opti-MEM and Lipofectamine 2000 (Invitrogen), in order to form complexes. RNAi complexes were added to 30% confluent N27 parental cells, which were equally plated in 60 mm dishes. Assays were performed 48 h after RNAi transfection.

### Immunoprecipitations

SUMOylation detection has been described as being highly susceptible to the action of proteases, therefore N-ethylmaleimide (NEM; 20 mM) and protease inhibitors were always present in cell and striatum lysates, and washing steps (Desterro et al., 1998; Martin et al., 2007; Tatham et al., 2011; Flotho and Melchior, 2013). In addition, we always performed immunoprecipitations using freshly extracted cell lysates to avoid losing SUMO-DAT reactivity. In our hands, sample freezing significantly reduced the SUMO-DAT signal and it was avoided. However, the extracted mouse striata were rapidly frozen and stored at −80°C, before homogenization and further assays. Protein concentration for each assay was quantified by the BCA assay (Thermo). Equal amount of protein was incubated with antibodies including rabbit anti-DAT (EL2, 1:1,000), rat anti-DAT (MAB, 1:1,000), rabbit anti-SUMO1 (EMD-Millipore 04-453, 1:1,000), mouse anti-ubiquitin (Santa Cruz sc-8017, 1:1,000), or control rabbit IgG (equivalent to the quantity used in experimental immunoprecipitation). Antibodies were added to freshly made cell lysates in RIPA buffer (plus 20 mM NEM and protease inhibitors) and incubated with rotation at 4°C overnight. On the following day, 60 μl of 50% protein-A Sepharose beads (GE Healthcare) for anti-DAT (EL2) antibody or protein-G Sepharose (Santa Cruz) for rat anti-DAT (MAB), were added to samples and incubated with rotation at 4°C for additional 2 h. Beads were extensively washed four times in cold RIPA buffer (plus 20 mM NEM and protease inhibitors). The resulting beads were suspended in 50 μl of Laemmli sample buffer containing 5% β-mercaptoethanol and incubated at 37°C for 30 min, prior to analysis using 4%–20% SDS-PAGE gels.

### Immunoblot Analyses

Samples solubilized in 1× Laemmli sample buffer, containing 5% β-mercaptoethanol were incubated at 37°C for 30 min, separated in 4%–20% MOPS SDS-PAGE gels, and transferred to PVDF membranes using the Bio-Rad system. PVDF membranes were first blocked in TBS-T buffer (50 mM Tris-HCl, 150 mM NaCl, and 0.2% Tween 20) containing 5% dry milk for 2 h and then incubated with the indicated primary antibody in the blocking buffer overnight, washed three times for 10 min each, and incubated with horseradish peroxidase-conjugated secondary antibodies (either goat anti-mouse, goat anti-rabbit, or goat anti-rat 1:5,000–10,000). Following all antibody incubations, membranes were washed three times in TBS-T buffer, and protein bands were visualized using the Prime Western blotting detection system (GE HealthCare). Primary antibodies include rabbit anti-TH (EMD-Millipore AB152, 1:1,000), rabbit anti-DAT “EL2” (EMD-Millipore AB5802, 1:1,000), rat anti-DAT “MAB” (EMD-Millipore MAB369, 1:1,000), mouse anti-SUMO1 (Santa Cruz sc-5308, 1:1,000), mouse anti-HA antibody (Santa Cruz sc-7392, 1:1,000), mouse anti-ubiquitin (Santa Cruz sc-8017, 1:1,000), rabbit anti-Ubc9 (Santa Cruz sc-10759, 1:1,000), and mouse anti- β-actin (EMD-Millipore MAB1501, 1:5,000). The PageRuler pre-stained protein ladder was used to estimate protein molecular weights on immunoblots (Thermo 26616).

### Densitometry

Immunoblot images were converted into 8-bit gray-scale images. Equal areas corresponding to selected lanes were analyzed on each blot image. Pixel values (0–255 scale) for each selected area, were obtained using ImageJ software and subtracted from the background. ECL PD was captured digitally in a highly sensitive and wide dynamic range apparatus (MyECL Imager, Thermo Scientific). Image saturation was always tested digitally in advance, and oversaturated images were avoided. Image splicing in panels was performed within a single larger image (splicing is indicated with thin gray bars). We did not put together spliced images that belonged to different experiments or dates. Splicing was implemented only for clarity purposes and the adjustment was performed using the original larger image.

### Exponential DAT Degradation Equations

For the exponential DAT decay in the N27 GFP cells, linear regression was calculated using Excel. The initial exponential DAT decay equation was fitted, producing *R*^2^ = 0.88. Improved curve fitting was achieved by plotting data in a semi-linear scale, obtaining a linear decay with *R*^2^ = 0.99: equation, *y* = −16.273*x* + 100.67. The calculated tau from the equation (37% of the previous value) was 49.4 h. The exponential DAT decay in the Ubc9-GFP cells was adjusted by linear regression and curve fitting using Excel, with *R*^2^ = 0.94: equation, *y* = 103.17 e-^0.012x^. The calculated tau from the equation (37% of the previous value) was 85.4 h.

### Chemicals

Phorbol 12-myristate 13-acetate (PMA, Sigma P1585) was used in the concentration of 1–10 μM. Twenty millimolar of NEM (Sigma E3876) and 100 μg/ml of cycloheximide was used (Sigma C7698). MG132 is Z-Leu-Leu-Leu-al, used in the concentration of 10 μM (Sigma C2211). Chloroquine diphosphate was used in the concentration of 10 μM (Sigma C6628). Protease inhibitor used was EDTA-free Halt protease inhibitor cocktail (Thermo Scientific 87785). 2-D08, a SUMOylation inhibitor III (Calbiochem 505156, EMD-Millipore) was used at the concentration of 6 μM. Transporter inhibitors in MPP^+^ uptake were included: GBR 12909 dihydrochloride (EMD-Millipore 505732), Mazindol (Sigma M2017), Desipramine hydrochloride (Sigma D3900), and Citalopram (Sigma C7861).

### Electrophysiology

Whole cell voltage clamp recording: HEK-DAT cells were plated on 105/35 mm culture dish. Attached cells were washed three times in external solution (130 mM NaCl, 10 mM HEPES, 34 mM dextrose, 1.5 mM CaCl_2_, 0.5 mM MgSO_4_, and 1.3 mM KH_2_PO_4_, adjusted to pH 7.35) at room temperature. The pipette solution for the whole cell recording contained a natural physiological-like internal solution containing 120 mM KCl, 0.1 mM CaCl_2_, 2 mM MgCl_2_, 1.1 mM EGTA, 10 mM HEPES, and 30 mM dextrose. Patch electrodes were pulled from quartz pipettes on a P-2000 puller (Sutter Instruments, Novato, CA, USA) and filled with the pipette solution. Whole-cell currents were recorded using an Axopatch 200B (Molecular Devices, Sunnyvale, CA, USA) with a low-pass Bessel filter set at 1,000 Hz. Inward currents were evoked by a series of hyperpolarization steps from −40 to −120 mV for 500 ms with 10 mV increments every 5 s from the holding membrane potential of −40 mV. Steady-state currents at 480 ms were measured and current-voltage (I-V) relationships were then plotted. The mean peak values of outward currents were plotted against membrane potential. Data were recorded and analyzed off-line using pCLAMP9 software (Molecular Devices). Before seals (5 Gohm) were made on cells, offset potentials were nulled. Capacitance subtraction was used in all recordings. Membrane potential measurements were not corrected for the liquid junction potential (~15 mV) and the series resistances were in the range of 10–20 MW.

### [^3^H]-DA Uptake Assay

HEK293-DAT cells were transfected with 5 μg of the corresponding cDNAs using Lipofectamine 2000 (Invitrogen), following the manufacturer’s instructions. The uptake assay was carried out at 37°C 48 h after the transfection. Cells were seeded (1–1.5 × 10^5^ cells per well) 24 h after transfection in a 24-well plate pre-coated with poly-D-Lysine. Before the experiment, cells were washed once with uptake buffer containing (in mM): 5 Tris base, 7.5 HEPES, 120 NaCl, 5.4 KCl, 1.2 CaCl_2_, 1.2 MgSO_4_, 0.1 ascorbic acid, 0.1 pargyline, 1 tropolone and 5 glucose, pH 7.4. Increasing concentrations of non-labeled dopamine (0, 0.1, 1, 3, 10, 20, and 30 μM) were added and the uptake was initiated by the addition of 0.02 μM of [^3^H]-dopamine (3,4-[7-^3^H] dihydroxy-phenylethylamine; 46 Ci/mmol; PerkinElmer), in a final volume of 0.4 ml. After 5 min, cells were washed in ice-cold NaCl-free uptake buffer, lysed with 0.4 ml 1% SDS, and the incorporated [^3^H]-dopamine was measured by a liquid scintillation counter (LSC 6000, Beckman Coulter). Non-specific uptake was determined with 0.01 mM GBR12909 (DAT inhibitor) or 1 mM dopamine. All experimental points were triplicated, expressed as mean ± SE, and independent transfections were considered as independent experiments. Uptake data were analyzed by non-linear regression analysis with SigmaPlot 12.5 (Systat Software).

### Mouse Striata Extraction

All animal protocols were conducted in accordance with the United States Public Health Service Guide for the Care and Use of Laboratory Animals; all procedures were approved by the Delaware State University Animal Care and Use Committee. All efforts were made to minimize animal numbers and distress. C57BL/6J male mice (9 months of age) were obtained from Charles River Laboratory (Wilmington, MA, USA). Mice were allowed *ad libitum* food and water and were housed five per cage. They were kept on a 12 h light-dark cycle and the food was normal mouse chow. For the striatum extraction, mice were fully anesthetized with isoflurane and then decapitated. The whole brain was isolated and striatum was carefully extracted under the surgical microscope, snap frozen with liquid nitrogen. Samples were homogenized in RIPA buffer with a douncer and a polytron homogenizer, incubated with rotation at 4°C for 1 h, followed by centrifuging at 16,400 *g* for 30 min at 4°C. The supernatant was collected, measured for protein concentration by the BCA assay, and used in subsequent experiments.

### Statistical Analyses

**Figure 3:**
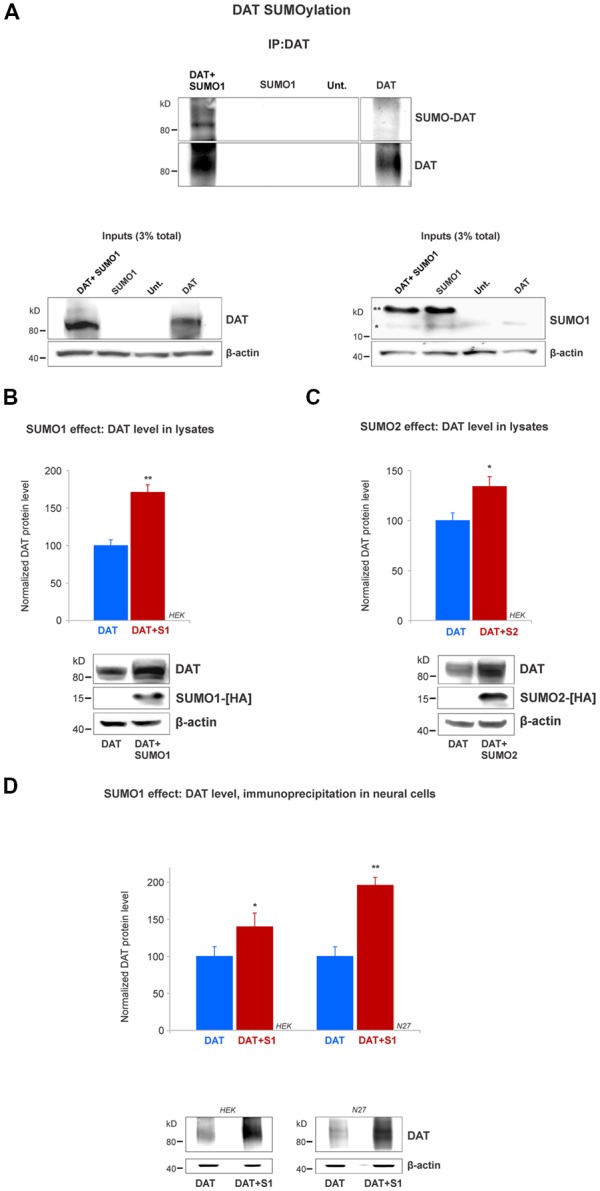
SUMO1 conjugated to DAT enhances DAT steady-state level. **(A)** HEK cells were transfected with DAT, DAT+SUMO1-HA, SUMO1-HA or left untransfected. DAT was immunoprecipitated with rabbit anti-DAT (EL2) and immunoblotted with anti-SUMO1 (mouse) or rat anti-DAT (MAB) antibody. SUMO1-DAT was identified as a characteristic smear with distinctive bands. Since HEK cells do not express DAT endogenously, we identified no SUMO1-DAT reactivity from untransfected (Unt.) or SUMO1 only transfected cells. Lysate inputs (3% total) are included at the bottom. DAT inputs are shown at the left and SUMO1 inputs are shown at the right. The free endogenous SUMO1 is indicated with a single star (*) and the overexpressed free SUMO1-HA is indicated with a double star (**). The SUMO1-HA migrates to a slightly higher level due to the presence of the HA tag. **(B)** SUMO1-HA overexpression increases the steady-state level of DAT. Either DAT alone or DAT+SUMO1-HA was transfected into HEK cells. Equal protein amounts were loaded and verified by probing β-actin. SUMO1-HA was detected with a mouse anti-HA antibody. DAT was identified by the anti-DAT (MAB) antibody (four independent experiments). **(C)** SUMO2-HA overexpression increases the protein level of DAT. DAT alone or DAT+SUMO2-HA was transfected into HEK. Equal protein amounts were loaded and verified by detecting β-actin. DAT was detected by the anti-DAT (MAB) antibody (three independent experiments) and SUMO2-HA was detected with mouse anti-HA antibody. **(D)** SUMO1 overexpression increases the protein level of DAT in N27 neural cells. DAT alone or DAT+SUMO1-HA was transfected into HEK or N27 cells. On each transfected cell line, equal amounts of protein lysate were immunoprecipitated by anti-DAT (EL2) and detected by anti-DAT (MAB) antibodies. β-actin was used as a protein loading control for DAT IPs. All values were normalized as average ± SE and statistical significance (*p < 0.05, **p < 0.01) was determined by a two-sided, Student’s *t*-test (three independent experiments).

**Figure 4:**
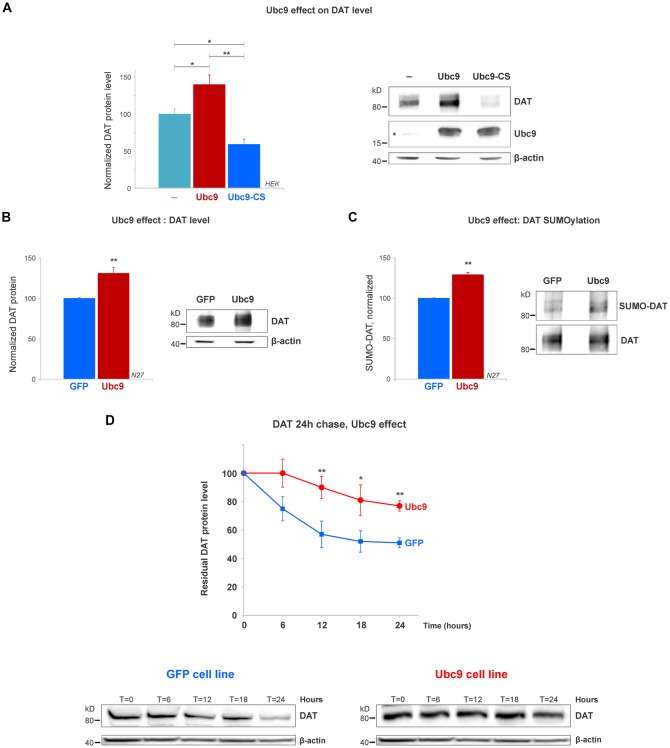
Ubc9 overexpression increases DAT steady-state levels by preventing DAT degradation in N27 and HEK cells. **(A)** The overexpression of Ubc9 induces higher DAT level than controls. HEK cells were transfected with DAT, DAT + Ubc9-HA, or DAT + Ubc9-CS-HA (inactive). DAT was detected by the anti-DAT antibody (MAB). Equal protein amounts were loaded and verified by probing β-actin. Ubc9 was detected by rabbit anti-Ubc9 antibody. Endogenous Ubc9 is indicated with a star. Statistical significance (*p < 0.05, **p < 0.01) was determined by one-way ANOVA with Tukey’s range test (results correspond to 7–14 independent experiments).** (B)** In the Ubc9-GFP and GFP N27 cell lines, Ubc9 overexpression significantly increased the level of endogenous DAT. DAT was immunoblotted with rabbit anti-DAT EL2 (six independent experiments). **(C)** The overexpression of Ubc9-GFP significantly increased the level of endogenous SUMO-DAT in N27 cells. DAT was immunoprecipitated with rabbit anti-DAT (EL2) and detected by anti-DAT (MAB) antibodies. SUMO-DAT was detected with mouse anti-SUMO1 antibody. The normalized SUMO-DAT value corresponds to the ratio SUMO-DAT/DAT (four independent experiments). **(D)** Ubc9-GFP decreases the endogenous DAT degradation rate in 24 h chase studies. N27 cells were incubated with cycloheximide and lysates were extracted at *T* = 0 (100% initial protein) and continued up to 24 h (*T* = 24 h). Cell extracts at the chase time point (*T* = 0, 6 h, 12 h, 18 h, and 24 h) were immunoblotted with anti-DAT (EL2). All values were normalized as averages ± SE. Statistical significance in DAT levels between Ubc9 and GFP was determined by a two-sided, Student’s *t*-test (*p < 0.05, **p < 0.01; three to four independent experiments).

**Figure 5:**
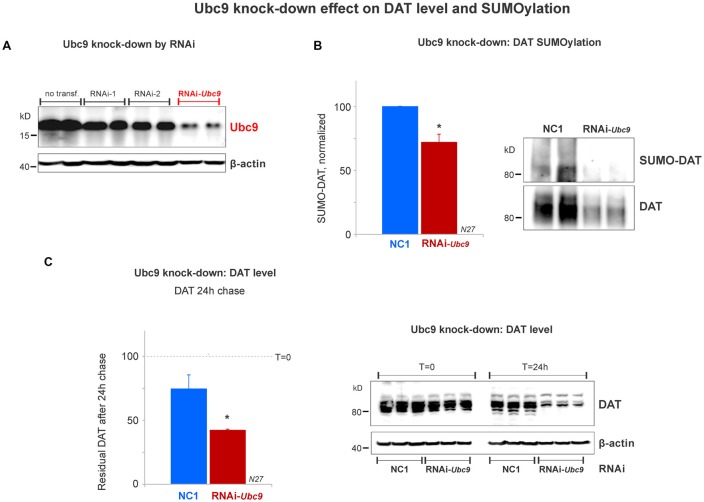
Ubc9 knock-down by interference RNA (RNAi) decreases DAT SUMOylation and enhances DAT degradation in N27 cells. **(A)** The combined RNAi-1 and -2 (RNAi-Ubc9) substantially reduced the level of endogenous Ubc9, compared with controls (no transfection or each individual RNAi construct). **(B)** Ubc9 knock-down by RNAi-Ubc9 decreases DAT SUMOylation when compared with NC1 (control). DAT was immunoprecipitated with anti-DAT (EL2) and detected by anti-DAT (MAB) antibodies, followed by the detection of SUMO-DAT with mouse anti-SUMO1 antibody. The normalized value of SUMO-DAT corresponds to the ratio of SUMO-DAT/DAT (three independent experiments). **(C)** In the 24 h chase study, Ubc9 knock-down reduces the residual level of endogenous DAT, which is derived from enhanced DAT degradation, compared with control (NC1). DAT was detected using anti-DAT (EL2) antibody (top) and the β-actin loading control is shown at the bottom. Values are presented as the average of the remaining fraction (*T* = 24 h) from the initial amount (100%, *T* = 0). Data represent mean ± SE. Statistical significance from normalized control (*p < 0.05) was determined by a two-sided, Student’s *t*-test (three independent experiments).

**Figure 6:**
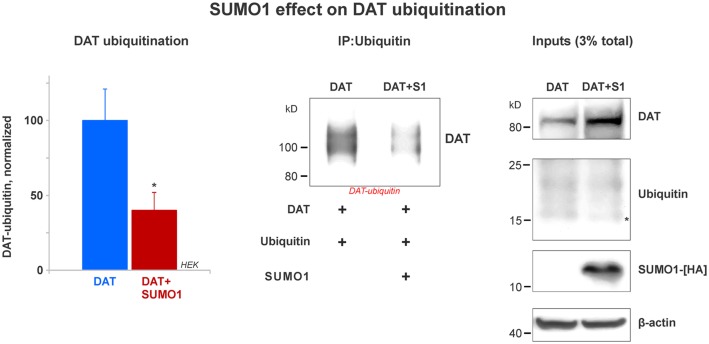
SUMO1 overexpression decreases DAT ubiquitination. HEK cells were transfected with DAT and ubiquitin to improve DAT-ubiquitin recovery. DAT-ubiquitin was immunoprecipitated by mouse anti-ubiquitin antibody in cells transfected with or without SUMO1-HA. Recovered DAT-ubiquitin was detected with anti-DAT (MAB) antibody. Inputs for DAT, ubiquitin, SUMO-HA are displayed. DAT was detected with anti-DAT (MAB) antibody. Di-ubiquitin is depicted with a star and it was detected with anti-ubiquitin (mouse) antibody. SUMO1-HA was detected with mouse anti-HA antibody. β-actin as a loading control is shown at the bottom. Data quantification from recovered DAT-ubiquitin represents mean ± SE and statistical significance from normalized control (*p < 0.05) was determined by a two-sided, Student’s *t*-test (four independent experiments).

**Figure 7:**
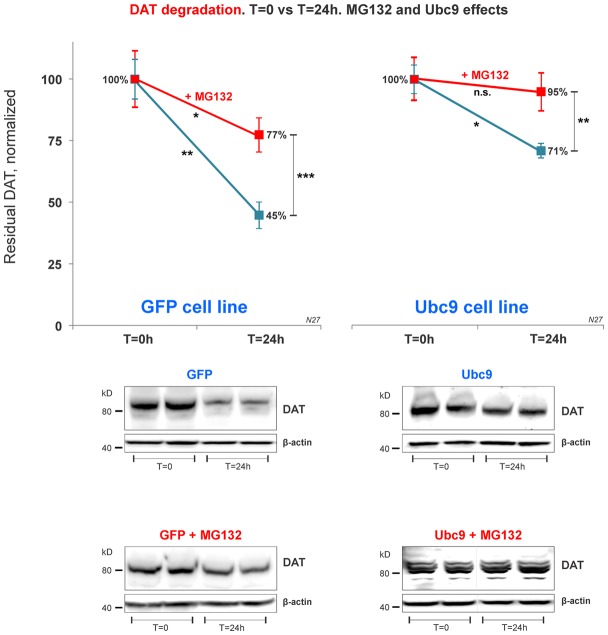
DAT proteasome-mediated degradation is independent of Ubc9 overexpression. In the cycloheximide 24 h chase study, N27 cell extracts were obtained at *T* = 0 (100% initial protein) and at 24 h (*T* = 24 h), with or without the presence of MG132 (proteasome inhibitor). Both cell extracts (*T* = 0 and *T* = 24 h) were immunoblotted with anti-DAT (EL2) antibody and re-probed with the anti β-actin antibody. Values are presented as an average of the remaining fraction at *T* = 24 h, compared with the initial normalized amount at *T* = 0 (100%). Data represent mean ± SE and statistical significance from control (*p < 0.05, **p < 0.01, ***p < 0.001, and n.s., not significant) was determined by a two-sided, paired, Student’s *t*-test (three to four independent experiments).

**Figure 8:**
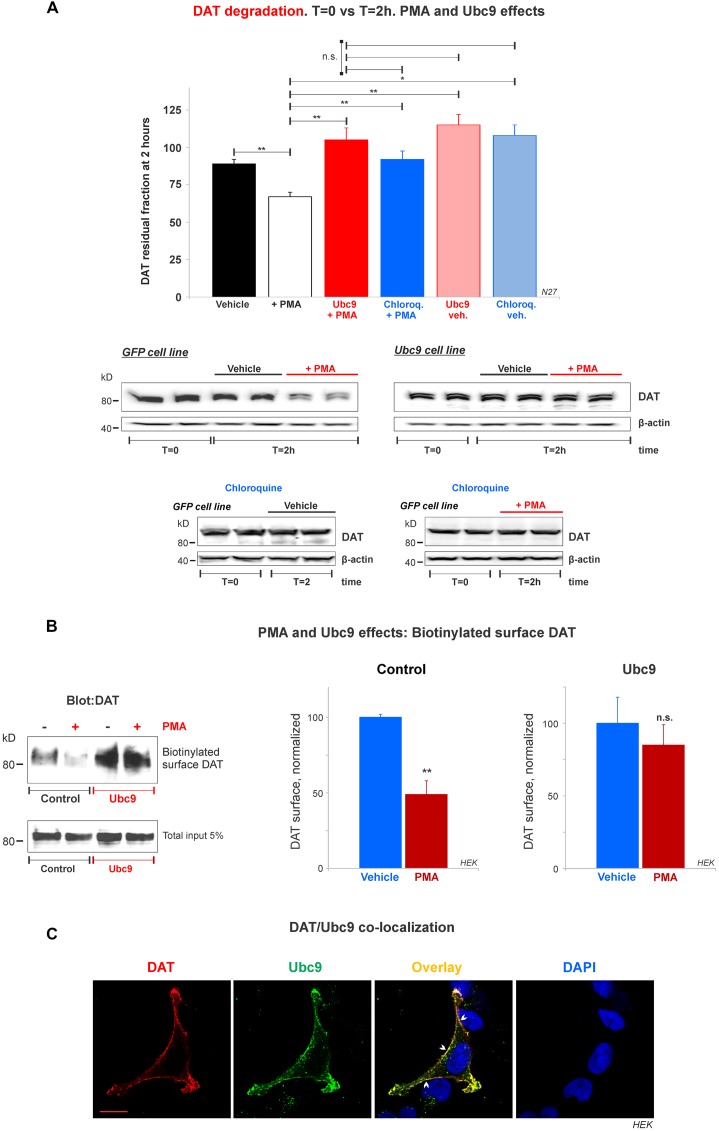
Ubc9 prevents Phorbol 12-myristate 13-acetate (PMA) induced DAT degradation in N27 cells. **(A)** Cycloheximide-treated 2 h chase studies with or without 2 μM PMA [protein kinase C (PKC) activator that induces DAT lysosomal degradation]. Residual DAT level was compared between *T* = 0 and *T* = 2 h, in the presence of chloroquine or vehicle. Both N27 cell extracts (*T* = 0 and *T* = 2 h) were immunoblotted with anti-DAT (EL2) antibody. Values are presented as the average of the remaining fraction at *T* = 2 h from the initial amount (100%, *T* = 0). Statistical significance (*p < 0.05, **p < 0.01) was determined by one-way ANOVA with Tukey’s range test (3–7 independent experiments).** (B)** Ubc9 blocks DAT internalization induced by PMA. HEK-DAT cells were incubated with vehicle or 1 μM PMA for 30 min. Cell surface biotinylation preceded using non-permeable sulfo-NHS-biotin and DAT was detected with anti-DAT (MAB) antibody. Values are subtracted from the background and displayed as average ± SE and statistical significance (**p < 0.01) was determined by a two-sided, Student’s *t*-test (three independent experiments).** (C)** DAT is co-localized with Ubc9 in the plasma membrane. Representative confocal microscopy images from permeabilized HEK-DAT cells expressing Ubc9-HA, immunostained with rat anti-DAT (MAB) or mouse anti-HA antibodies and secondary antibodies conjugated with Alexa 546 (red) or Alexa 488 (green), respectively (pseudocolor was reversed for consistency). DAPI (blue) was used for nuclear staining (*n* = 3). Bar: 5 μm. ns, not significant.

**Figure 9:**
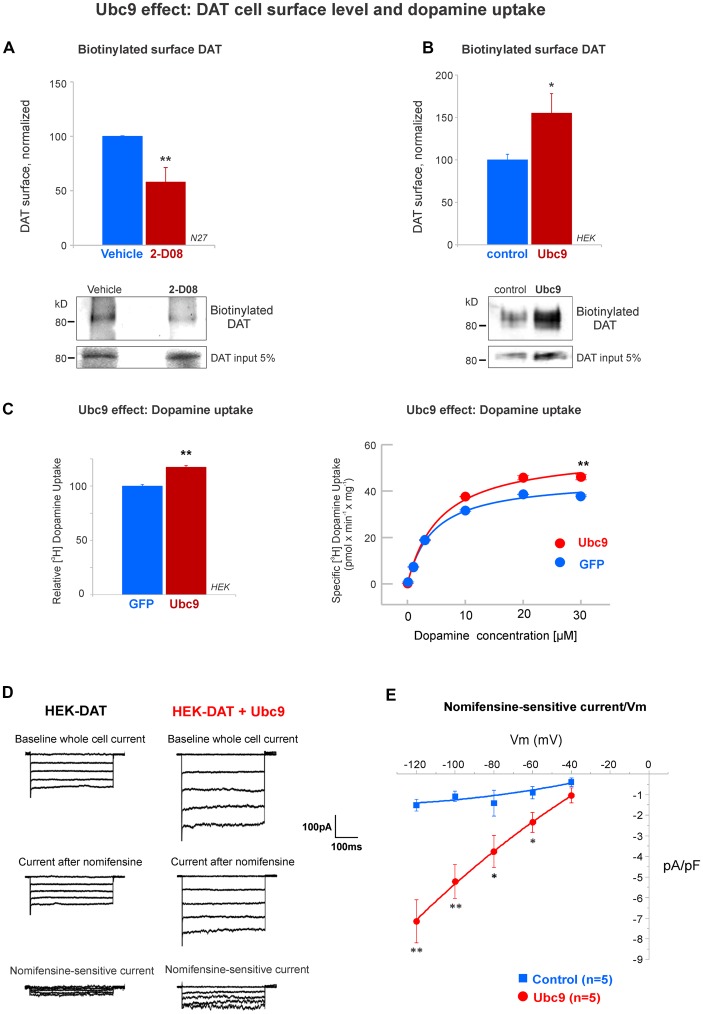
Ubc9 enhances the functional level of DAT in the plasma membrane. **(A)** The Ubc9 inhibitor 2-D08 significantly reduces the surface level of DAT in N27 cells. Surface biotinylated DAT was immunoblotted with anti-DAT (MAB) antibody, including DAT 5% input (four independent experiments). **(B)** Surface biotinylated DAT level was significantly increased with Ubc9 overexpression in HEK-DAT cells. Values were normalized as average ± SE (six independent experiments). **(C)** Dopamine uptake is enhanced by Ubc9-GFP overexpression in HEK-DAT cells. Left, Ubc9 increases the maximum uptake of relative 3,4-[7-^3^H] dihydroxy-phenylethylamine ([^3^H]-dopamine) in comparison to GFP control cells. Non-specific dopamine uptake was determined in the treatment of 0.01 mM GBR12935 or 1 mM dopamine (five independent experiments). Right, representative saturation curve of [^3^H]-dopamine uptake in HEK293-DAT cells transfected with GFP or Ubc9-GFP. The overexpression of Ubc9 resulted in an increase in the maximum specific dopamine uptake velocity without a change in the affinity for dopamine. **(D)** Ubc9-GFP increases DAT-mediated uncoupled currents. HEK-DAT cells were voltage clamped in the whole cell configuration, 24–36 h after transfection, while the membrane potential was stepped from −120 to −40 mV from a holding potential of −40 mV in 20 mV increments. Representative traces of whole cells and nomifensine-sensitive uncoupled DAT currents are shown (three independent experiments). **(E)** Nomifensine sensitive currents were plotted in a current-voltage relationship. All values are shown as average ± SE and statistical significance (*p < 0.05, **p < 0.01) was determined by a two-sided Student’s *t*-test.

In most statistical analyses, unpaired Student’s *t*-test was applied to assess the effect of drug treatment or Ubc9/SUMO/ubiquitin overexpression on DAT levels when compared with controls: Figures 2C, 3B–D, 4B–D, 5B,C, 6, 7, 8B, 9A–C,E, Supplementary Figures S3, S6. Additional two-way ANOVA was adopted to assess the interaction effect between MG132 and Ubc9 (Figure 7). In Figures 1D, 2D, 4A, 8A, the comparison was performed by applying one-way ANOVA, Tukey’s *post hoc* test. For all studies, *p* < 0.05 was considered statistically significant (*); GraphPad Prism 5 and Excel software were used for all data analyses and display, as previously described (Lazzara et al., 2015).

## Results

### DAT Is Endogenously Expressed in Dopaminergic N27 Cells

In order to study DAT SUMOylation in a natural environment *in vitro*, we chose the immortalized rat dopaminergic N27 cell line which has been characterized as a suitable dopaminergic model in previous studies (Adams et al., 1996; Clarkson et al., 1998). The N27 cells express TH, DAT, and neuronal markers. They synthesize dopamine and mature into functional dopaminergic neural cells upon transplantation in animals (Adams et al., 1996; Clarkson et al., 1998). However, a thorough characterization of endogenous DAT expression and function has not been implemented in N27 cells. In Figure 1A, endogenous DAT was identified as a band of around 82kD by immunoblotting of N27 cell lysates with the well-characterized rat anti-DAT antibody (MAB). Non-transfected HEK cells do not express DAT and were used as negative controls. Transfected DAT in HEK cells (HEK-DAT) was used as a positive control for the MAB antibody. Next, we demonstrated by immunofluorescence and confocal microscopy that DAT was endogenously expressed in fixed N27 cells which also express TH to synthesize dopamine (Figure 1B). The secondary antibody-only images with DAPI nuclear staining were displayed as negative controls to support the specific label of DAT and TH. We also performed N27 cell surface biotinylation assays that were differentially immunoblotted with the anti-DAT EL2 (rabbit) or anti-DAT MAB antibodies, demonstrating that DAT is localized to the cell surface. Here, calnexin, an ER marker, was used as a negative control to demonstrate that the N27 cell surface integrity remained intact during the biotinylation procedure (Figure 1C). We then tested whether the DAT present in the plasma membrane was functional, measuring specific MPP^+^ uptakes. Since MPP^+^ is known to be taken up by DAT and related transporters such as NET and SERT, we applied four different uptake inhibitors: mazindol (high affinity for NET and DAT), GBR12935 (high affinity for DAT), desipramine (high affinity for NET), and citalopram (high affinity for SERT; Figure 1D and Table 1; Hyttel, 1982; Eshleman et al., 1999). As shown in Figure 1D, MPP^+^ uptake was significantly inhibited by the presence of the DAT blockers, GBR12935 or mazindol, in addition to marginal inhibition by desipramine, suggesting that DAT is active and a major transporter to uptake MPP^+^ in N27 cells (normalized MPP^+^ uptake: no inhibitor = 100% ± 1.7; mazindol = 28.6% ± 2.6; GBR12935 = 43.8% ± 5.2; desipramine = 80.3% ± 3.6; citalopram = 83.5 ± 8.1). Overall, these results demonstrate that endogenous DAT is expressed in the N27 cells and still functional and active in the plasma membrane.

### DAT Associates With SUMO1 in FRET Studies

A number of reports have demonstrated that SUMO can associate with its target proteins non-covalently (Tang et al., 2005; Dustrude et al., 2013; Flotho and Melchior, 2013; Henley et al., 2014). This possibility was investigated in HEK-DAT cells transfected with SUMO1-HA. HEK-DAT cell extracts were immunoprecipitated with rabbit anti-SUMO1 or IgG antibodies (negative control). Our results showed that DAT co-precipitates with SUMO1-HA. The DAT was identified in the 10% input corresponding to the lysate, prior to immunoprecipitations. This result suggests that SUMO1 can associate with DAT (Figure 2A).

In order to study the DAT and SUMO1 association by FRET in intact cells, we produced a stable N27 cell line overexpressing DAT-YFP. FRET between DAT-YFP and transiently transfected SUMO1-CFP was performed using the acceptor photobleaching method (Karpova et al., 2003; Karpova and McNally, 2006). If two proteins are associated, FRET acceptor photobleaching allows one to detect a positive increment in the donor fluorescence (SUMO1-CFP) when the acceptor fluorescence (DAT-YFP) is photobleached. In Figure 2B, a full spectrum is shown in the histogram of a typical experiment; photobleaching of the FRET acceptor DAT-YFP (peak fluorescence at 530 nm) determined a positive increase in the fluorescence of the FRET donor SUMO1-CFP (peak fluorescence at 477 nm). Confocal microscopy images of the experiment are shown at the bottom, featuring images of the pre- and post-DAT-YFP photobleaching and the increase in SUMO1-CFP fluorescence after DAT-YFP bleaching (see “Materials and Methods” section). Quantification of the peak fluorescence before and after photobleaching is shown in Figure 2C. The SUMO1-CFP fluorescence increased after DAT-YFP photobleaching (normalized YFP-DAT fluorescence at 530 nm: pre-bleaching = 100% ± 13, post-bleaching = 23% ± 10; SUMO1-CFP fluorescence at 477 nm: pre-bleaching = 100% ± 9, post-bleaching = 119% ± 10; Figure 2C). Since FRET efficiency is the most important parameter to determine FRET specificity between DAT-YFP and SUMO1-CFP, we performed a number of control experiments as shown in Figure 2D and Table 2. The FRET efficiency of DAT-YFP + SUMO1-CFP (#3 in Figure 2D) was significantly lower than the positive control, tandem YFP-CFP (#1 in Figure 2D), but higher than the negative controls including YFP + CFP (Table 2), DAT-YFP + CFP (#4), YFP + SUMO1-CFP (#5), SUMO1-CFP alone (#6), and non-photobleached regions on DAT-YFP + SUMO1-CFP (#7 in Figure 2D; see “Materials and Methods” section for procedures). On the other hand, the FRET efficiency of the well-characterized DAT-YFP/DAT-CFP interactive pair (#2 in Figure 2D) was not significantly different from the DAT-YFP/SUMO1-CFP pair. Altogether our results demonstrate that DAT and SUMO1 can be physically associated.

### SUMO1 Conjugates and Enhances DAT Steady-State Level

Since we demonstrated that DAT and SUMO1 were physically associated, we next tested whether SUMO1 was conjugated to DAT. The DAT structure contains multiple potential lysine residues that can be targeted for SUMOylation (Miranda et al., 2005, 2007; Rudnick et al., 2014; German et al., 2015). HEK cells were transiently transfected with DAT alone, SUMO1-HA alone, or SUMO1-HA+DAT. HEK cell lysates were immunoprecipitated with anti-DAT (EL2) antibodies and detected by anti-DAT (MAB) antibodies (Figure 3A). DAT-SUMO1 was identified by the anti-SUMO1 antibody (mouse) as a distinctive smear with noticeable bands when both DAT and SUMO1 were overexpressed (Figure 3A, top). As many studies have demonstrated, it is a common feature to detect smear bands of modified proteins conjugated to one or more ubiquitin-like molecules such as SUMO (Miranda et al., 2005, 2007; Yan et al., 2005; Tatham et al., 2011; Atkin and Paulson, 2014). The DAT-SUMO1 bands were not detected when DAT was not transfected, or when SUMO1-HA was transfected alone to HEK cells (Figure 3A, top). The presence of DAT and SUMO1-HA was detected in the input lysates (Figure 3A, bottom). Free endogenous SUMO1 (*) and SUMO1-HA (**) migrate differentially due to the presence of the HA-tag on the transfected SUMO1-HA (Figure 3A, bottom right). Although endogenous SUMO1 is expressed in HEK cells (Figure 3A, bottom right), SUMO-DAT was barely detectable without DAT+SUMO1-HA overexpression (Figure 3A, top).

Remarkably, we observed that transient transfections of DAT together with SUMO1-HA increased the protein level of DAT (Figure 3A). Therefore, we performed a quantification of the DAT steady-state level between DAT co-transfected with or without SUMO1-HA in HEK cells. As shown in Figure 3B, the co-expression of SUMO1-HA induced a higher DAT steady-state level when detected by the anti-DAT (MAB) antibody (DAT only = 100% ± 8, DAT+SUMO1-HA = 171% ± 10; Figure 3B). The presence of overexpressed SUMO1-HA was detected by anti-HA antibody and β-actin was used as an equal loading control. The increase in the DAT steady-state level by SUMO1-HA was replicated when we co-expressed DAT with the other SUMO paralogue, SUMO2-HA. As displayed in Figure 3C, the co-expression of SUMO2-HA also induced an increased DAT steady-state level when detected by anti-DAT (MAB) antibody (DAT only = 100% ± 16, DAT+SUMO2-HA = 134% ± 18; Figure 3C). The overexpressed SUMO2-HA was detected by anti-HA antibody and β-actin blotting was used as a loading control. As shown in Figure 3D, to reproduce the observed SUMO-dependent increase in DAT level, in dopaminergic N27 cells, we performed parallel immunoprecipitations in transfected HEK and N27 cells. Transfected DAT was immunoprecipitated by anti-DAT (EL2) and detected by anti-DAT (MAB) antibodies in both the N27 and HEK cells (HEK cells: DAT only = 100% ± 13, DAT+SUMO1 = 146% ± 19; N27 cells: DAT only = 100% ± 10.6, DAT+SUMO1 = 196% ± 13, Figure 3D). β-actin was used as an equal loading control for immunoprecipitations. Overall, these results strongly suggest that SUMO overexpression enables DAT to conjugate and associate with SUMO1 and also significantly increases the DAT steady-state level.

In addition, in order to assess whether DAT was a substrate for SUMO1 in the mouse brain, we performed experiments using isolated mice striatum, an area of the brain containing the highest number of DAT positive terminals. DAT SUMOylation in the brain was demonstrated by immunoprecipitation with the anti-SUMO1 antibody (rabbit) to recover the SUMO-DAT species (SUMO-DAT, Supplementary Figure S1). As suitable controls, we performed immunoprecipitations with the anti-DAT antibody (EL2, rabbit) and generic IgG (rabbit). The immunoprecipitation with anti-SUMO1 antibody recovered SUMO-DAT, which was detected as a high molecular weight (HMW) smear featuring distinctive bands (SUMO-DAT, indicated with a bracket in Supplementary Figure S1). We detected the recovered SUMO-DAT species with the anti-DAT antibody (MAB). The identified SUMO-DAT appears to be compatible with SUMO1 conjugated to DAT at one or multiple residues. However, SUMO-DAT seems to correspond to only a small fraction of the total-DAT present in the striatum (total-DAT, Supplementary Figure S1). After immunoprecipitation with the anti-DAT antibody, the recovered DAT showed a tendency to migrate at a lower molecular weight level (total-DAT, Supplementary Figure S1).

The total DAT corresponds to both SUMOylated and non-SUMOylated DAT species. However, the DAT fraction migrating as a HMW smear was “almost” undetectable. This is in line with many communications that only a small fraction of total protein (about a 5%) is SUMOylated at any given time (Hay, 2005; Flotho and Melchior, 2013; Henley et al., 2014). We also detected the presence of SUMOylated species in the striatum, where free SUMO was identified as a protein of ~15kD (Supplementary Figure S1, right panel, indicated with a star). Overall, our results strongly suggest that DAT can be SUMOylated by SUMO1 in both cellular *in vitro* systems and dopaminergic terminals in the striatum.

### Ubc9 Increases the DAT Steady-State Level by Reducing DAT Degradation Rate

The SUMO conjugase Ubc9 is the single enzyme that transfers SUMO1 and SUMO2 to target proteins. To conjugate SUMO to its targets, Ubc9 interacts with proteins through a specific cysteine residue (C93), however, when it is mutated, inactive Ubc9 acts as a dominant negative for the endogenous Ubc9 (Hayashi et al., 2002; Hay, 2005; Flotho and Melchior, 2013; Gupta et al., 2014; Henley et al., 2014). Therefore, we tested whether Ubc9 overexpression increased the DAT steady-state level. As shown in Figure 4A, the overexpression of Ubc9 in HEK cells significantly increased the DAT level when compared with the untransfected controls, while the inactive Ubc9 C93S (Ubc9-CS) significantly reduced the DAT protein level (DAT level, Control = 100% ± 7; Ubc9 = 140% ± 13; Ubc9-CS inactive = 59% ± 7, Figure 4A). The presence of Ubc9 was detected by the anti-Ubc9 antibody (rabbit). The transfected Ubc9 species migrated differentially from endogenous Ubc9 due to the presence of an HA-tag (the endogenous Ubc9 is indicated with a star in Figure 4A Ubc9 blot). β-actin detection was used as an equal loading control.

Next, using the N27 cells as a suitable dopaminergic neural model, we developed two stable N27 cell lines, overexpressing Ubc9 tagged with GFP (Ubc9-GFP) or GFP only (used as a control), to test whether Ubc9-induced SUMOylation affects DAT steady-state level, degradation, and functional expression in the plasma membrane. In Figure 4B, the endogenous DAT steady-state level was significantly higher in Ubc9-overexpressing cells than that in GFP controls (normalized DAT values, Ubc9-GFP = 131% ± 7.3; GFP = 100% ± 1; Figure 4B). These results were similar to those obtained in HEK cells transfected with Ubc9 (Figure 4A). Next, we compared the level of DAT conjugation by endogenous SUMO1 in the Ubc9-GFP with that in GFP cells. In SUMO1-DAT quantification, we demonstrated that the SUMO1-DAT level was significantly higher in the Ubc9-GFP cells than that in the GFP cells (normalized SUMO1-DAT: Ubc9-GFP = 129% ± 2, GFP = 100% ± 1; Figure 4C). We then tested whether the increase of DAT steady-state level in Ubc9-GFP cells was derived from the decrease in DAT degradation. We performed DAT chase analysis using the protein synthesis inhibitor cycloheximide to compare the recovered amount of DAT in Ubc9-GFP with that in GFP cells in 24 h chase analyses. The degradation rate of DAT was significantly decreased in Ubc9-GFP cells, compared to GFP cells (recovered DAT at 24 h: Ubc9-GFP = 77% ± 4, GFP = 51% ± 3; Figure 4D). Additional examples of the differences between GFP and Ubc9-GFP cells were shown in Supplementary Figure S2. We adjusted each DAT degradation curve with an exponential decay equation (see “Materials and Methods” section) and calculated the time constant (tau) of DAT degradation rate for each cell line: GFP, tau = 49 h and Ubc9-GFP, tau = 85 h. Thus, the time constant for DAT degradation was significantly higher in the Ubc9-GFP cells than that in GFP cells, indicating that DAT became a more stable protein. Since Ubc9 and SUMO have been described as regulators for nuclear trafficking and transcription factors, we also tested the possibility that Ubc9-GFP upregulated DAT transcription, which could explain the enhanced DAT level (Hayashi et al., 2002; Lee et al., 2011; Flotho and Melchior, 2013; Henley et al., 2014). Therefore, we performed qRT-PCR for DAT, using β-actin as a housekeeping gene. In order to examine the differences in the ratio of DAT crossing point (C_p_) values (the cycle number of a gene’s initial amplification point), we measured the ratio of DAT/β-actin, as determined by fluorescence of SYBR-green. Although DAT protein level was enhanced in the Ubc9-GFP cells, our qRT-PCR data revealed no difference in the level of DAT mRNA between GFP and Ubc9-GFP cells (ratio of DAT/β-actin, GFP = 1.05 ± 0.03; Ubc9-GFP = 1.04 ± 0.01; Supplementary Figure S3). This result demonstrates that the enhanced DAT protein expression in the Ubc9-GFP cell line was not due to the increase of DAT mRNA level.

Altogether our data indicate that Ubc9 overexpression increases DAT SUMOylation and enhances the DAT steady-state level by preventing the degradation of DAT without affecting DAT transcription or synthesis.

### Ubc9 Knock-Down Reduces DAT SUMOylation and Increases DAT Degradation

Since the SUMOylation of DAT reduced DAT degradation, we tested whether DAT degradation was increased when endogenous Ubc9 expression was down-regulated by Ubc9 RNAi in N27 cells. We titrated two different RNAi constructs that targeted Ubc9 mRNA (RNAi-1 and RNAi-2). The optimal Ubc9 down-regulation was obtained by the combination of 80 pmoles of each RNAi (RNAi-Ubc9, Figure 5A). Once the optimal RNAi conditions for knocking down the endogenous Ubc9 were established, we identified that SUMOylated DAT (SUMO-DAT) was significantly reduced in N27 cells transfected with the RNAi-Ubc9 mix (normalized SUMO-DAT: NC1 = 100% ± 0.2; RNAi-Ubc9 = 72% ± 10, Figure 5B). In Figure 5C, the down-regulation of endogenous Ubc9 with the RNAi-Ubc9 mix significantly increased DAT degradation in the cycloheximide chase studies. The residual level of DAT in transfected cells with RNAi-Ubc9 mix was significantly reduced after 24 h of DAT chase, compared with the negative control treatment, NC1 (recovered DAT at 24 h: NC1 = 75% ± 11; RNAi-Ubc9 = 42% ± 1; Figure 5C). In addition, we routinely observed an evident down-regulation of DAT 1 day after RNAi mix transfection, when compared with the beginning of the cycloheximide chase (*T* = 0, Figure 5C). Our results demonstrate that the reduction in DAT SUMOylation by Ubc9 knock-down increases DAT degradation. Together with the results in Figure 4, our data strongly suggest that DAT steady-state level is directly dependent on its association with SUMO and DAT SUMOylation level.

### SUMO1 Overexpression Decreases DAT Ubiquitination

Many reports have demonstrated that DAT modification by ubiquitin targets DAT for lysosomal degradation (Daniels and Amara, 1999; Miranda et al., 2005, 2007; Hong and Amara, 2013). DAT ubiquitination involves the addition of short polyubiquitin chains, increasing about 20–25 kD in the DAT molecular weight (Daniels and Amara, 1999; Miranda et al., 2005, 2007; Hong and Amara, 2013). Recent studies have demonstrated an important cross-talk between the SUMO and ubiquitin pathways that determines the steady-state level of relevant proteins such as α-synuclein and Huntingtin (Steffan et al., 2004; Tatham et al., 2008, 2011; O’Rourke et al., 2013; Liebelt and Vertegaal, 2016; Rott et al., 2017; Rousseaux et al., 2018). Here, we tested whether SUMO1 overexpression decreased DAT ubiquitination. HEK cells were transfected with both ubiquitin and DAT with or without the overexpression of SUMO1-HA. The overexpression of ubiquitin allowed us to recover a measurable quantity of DAT-ubiquitin and determine the effect of SUMO1 on DAT ubiquitination levels. Ubiquitin overexpression did not result in a decrease on the DAT steady-state level when compared with cells without ubiquitin transfection (data not shown). As shown in Figure 6, we compared the level of DAT ubiquitination in transfected HEK cells with or without SUMO1-HA by immunoprecipitating DAT-ubiquitin with an anti-ubiquitin antibody (mouse). In DAT-ubiquitin quantification, the ubiquitination level of DAT was significantly reduced when SUMO1-HA was overexpressed (DAT-ubiquitin recovered: Control = 100% ± 21; SUMO1-HA = 40% ± 12; Figure 6). In addition, when either SUMO1-HA or SUMO2-HA was transfected, we observed that there was also a substantial decrease in the level of DAT-ubiquitin recovered (Supplementary Figure S4). Inputs display that the levels of ubiquitin were similar between SUMO-HA + DAT overexpression and DAT only. However, the DAT steady-state level was obviously enhanced in the SUMO-HA overexpressing cells as expected (Figure 6 and Supplementary Figure S4). Overall, these results suggest that SUMO1 by its association with DAT enables to delay DAT degradation, as shown in Figures 4, 5 by decreasing DAT ubiquitination. Our results point out to a possible cross-talk between the SUMO and ubiquitin pathways to regulate the DAT steady-state level, which may be similar to that described for α-synuclein and Huntingtin. Since ubiquitin targets proteins for degradation *via* the proteasome and the lysosome pathways, we assessed whether the observed DAT degradation delay was due to a direct effect of Ubc9/SUMO on those pathways.

### DAT Proteasomal Degradation Is Independent of Ubc9 Overexpression

Recent studies have identified a role for SUMO in protein quality control in the endoplasmic reticulum (Hay, 2005; Tatham et al., 2008, 2011; Ahner et al., 2013; Gupta et al., 2014). Here, we tested whether DAT steady-state increase induced by Ubc9/SUMO was mediated by the inhibition of DAT proteasomal degradation pathway. After 24 h exposure of the proteasome inhibitor MG132 in the cycloheximide chase studies, the residual DAT level was measured in both Ubc9-GFP and GFP N27 cell lines. We measured the residual level of DAT in N27 cells between *T* = 0 and *T* = 24 h for each condition. Our results demonstrate that DAT degradation was significantly reduced in both cell lines when the proteasomal activity was blocked by MG132 (Figure 7). In our analysis, the Ubc9-GFP alone reduced DAT degradation by the same level of the MG132 proteasomal inhibitor in the GFP control cells. Importantly, the effect of Ubc9-GFP on the increase of DAT steady-state level appeared to be additive to that of MG132, since their combined effect almost completely blocked DAT degradation. In the analyses of paired *t*-test between *t* = 0 and *t* = 24 h, we found significantly reduced DAT degradation by MG132, Ubc9, and both treatments [residual DAT at 24 h: GFP without treatment = 45% ± 5.3 (*p* < 0.01); GFP+MG132 = 77% ± 7 (*p* < 0.05); Ubc9-GFP without MG132 = 71% ± 2.8 (*p* < 0.05); Ubc9-GFP+MG132 = 95% ± 7.8 (n.s.); Figure 7]. To determine whether the Ubc9 and MG132 effects were interrelated and additive, we statistically assessed the interaction effect between MG132 and Ubc9 using two-way ANOVA, Bonferroni *post hoc* and found that their statistical interaction was not significant (*p* = 0.081, *F* = 2.286). Our results suggest that the proteasome can be a major source for DAT degradation. However, our analysis support that the Ubc9 activity may have an additive capacity to reduce DAT degradation outside of the proteasomal pathway.

### Ubc9 Blocks DAT Lysosomal Degradation Induced by PMA and Co-localizes With DAT in the Plasma Membrane

Many studies have demonstrated that DAT in the plasma membrane can be regulated by PKC in a balance of endocytosis, recycling, and lysosomal degradation (Daniels and Amara, 1999; Miranda et al., 2005, 2007; Sorkina et al., 2005; Hong and Amara, 2013; Wu et al., 2015). Therefore, we tested whether Ubc9-GFP overexpression in N27 cells had an impact on PKC-induced DAT degradation. First, we assessed the capacity of Ubc9 to impair PMA-induced DAT degradation by lysosomes. PMA is considered a PKC activator and has been extensively used to study DAT turnover (Daniels and Amara, 1999; Miranda et al., 2005, 2007; Sorkina et al., 2005; Hong and Amara, 2013; Wu et al., 2015). We performed cycloheximide chase analyses in the presence of PMA with or without chloroquine, a known lysosomotropic agent that reduces lysosomal protease activities (Daniels and Amara, 1999). In Figures 8A and Supplementary Figure S5, we used 2 μM PMA for 2 h to induce DAT lysosomal degradation in N27-GFP cells. The PKC activation by PMA induced a significant DAT degradation, compared to vehicle only (residual DAT: vehicle = 89% ± 3; PMA = 67% ± 3; Ubc9+PMA = 105% ± 8; chloroquine+PMA = 92% ± 6; Ubc9-vehicle = 115% ± 7; chloroquine-vehicle = 108% ± 7, Figures 8A and Supplementary Figure S5). The DAT lysosomal degradation induced by PMA was blocked by co-incubation with the lysosomal proteases inhibitor, chloroquine. Importantly, we found that Ubc9-GFP overexpression prevented PMA-induced lysosomal degradation (Figures 8A and Supplementary Figure S5).

Since the DAT lysosomal degradation induced by PMA has been shown to be related to DAT internalization (Daniels and Amara, 1999; Miranda et al., 2005, 2007; Sorkina et al., 2005; Hong and Amara, 2013; Wu et al., 2015), we tested whether Ubc9 overexpression reduced DAT internalization. HEK-DAT cells were treated with 1 μM PMA for 30 min (control, Figure 8B). The cell surface DAT was biotinylated and detected by the anti-DAT (MAB) antibody to assess the DAT internalization triggered by PMA (normalized surface DAT control: PMA-treated = 49% ± 12; Vehicle = 100% ± 2, Figure 8B). Our results are compatible with the previous reports that PMA treatment promoted significant DAT internalization in heterologous systems (Daniels and Amara, 1999; Miranda et al., 2005, 2007; Sorkina et al., 2005; Hong and Amara, 2013; Wu et al., 2015). On the other hand, when HEK-DAT cells were transfected with Ubc9-HA, PMA treatment showed no significant DAT internalization, compared with untreated cells (normalized surface DAT in Ubc9: PMA-treated = 85% ± 14; Vehicle = 100% ± 14, Figure 8B). These results suggest that Ubc9 overexpression may block or delay DAT internalization induced by PMA in HEK-DAT cells.

To reinforce the notion that Ubc9 may exert part of its effects by modulating DAT internalization and degradation at the plasma membrane, we determined whether Ubc9 was targeted to the plasma membrane where it can be associated with DAT. HEK-DAT cells were transfected with Ubc9-HA, fixed, and permeabilized with DAT or Ubc9 antibodies (Figure 8C). The corresponding distribution of Ubc9 (Figure 8C, green), showed that Ubc9 co-localized with DAT (Figure 8C, red) in the plasma membrane (indicated by arrowheads in Figure 8C, overlay). These results suggest that Ubc9 can associate with DAT in the plasma membrane where DAT is physiologically active and relevant.

In particular, Ubc9 has been described to localize in the nuclear and perinuclear regions where it is available to regulate signaling pathways in the cell nucleus (Hayashi et al., 2002; Lee et al., 2011; Flotho and Melchior, 2013; Henley et al., 2014). In our observation, overexpressed Ubc9 was also substantially present in the nucleus and nuclear periphery of the HEK-DAT and Ubc9-GFP cell lines (data not shown). Therefore, the Ubc9 expression at the plasma membrane appears to be a singularity, which has been reported when membrane proteins are regulated by the Ubc9/SUMO pathway (Giorgino et al., 2000; Rajan et al., 2005; Liu et al., 2007; Benson et al., 2017).

Our combined results in Figure 8 suggest that Ubc9 can reach the plasma membrane and associate with DAT, delaying or preventing DAT internalization and degradation by lysosomes in HEK-DAT and dopaminergic N27 cell lines.

### Ubc9 Enhances DAT Functional Expression in the Plasma Membrane

The functional activity of DAT on the cell surface is the most important factor for dopamine clearance (Daniels and Amara, 1999; Loder and Melikian, 2003; Miranda et al., 2005, 2007; Sorkina et al., 2005; Hong and Amara, 2013; Rudnick et al., 2014; German et al., 2015; Wu et al., 2015). Since we demonstrated that Ubc9 reduced PKC-mediated DAT degradation *via* lysosomes, we next tested if Ubc9 activity affected DAT function on the cell surface. First, we tested the effect of pharmacological inhibition of Ubc9 with 2-D08 on the cell surface level of endogenous DAT in N27 cells. The compound 2-D08 has been recently characterized as a cell-permeable inhibitor for blocking the transfer of SUMO1 from Ubc9 to the substrate (Kim et al., 2013). We incubated the N27 cells with 6 μM 2-D08 for 48 h, as 6 μM corresponds to the IC50 for 2-D08 (Kim et al., 2013). As shown in Figure 9A, 2-D08 significantly reduced the level of DAT on the cell surface when biotinylated surface proteins were assessed (surface DAT: vehicle = 100% ± 0.4; 2-D08 = 58% ± 13; Figure 9A). Next, the effect of Ubc9 overexpression was tested in HEK-DAT cells and surface DAT level was measured by surface biotinylation (vector: normalized DAT = 100% ± 7; Ubc9: normalized surface DAT = 155% ± 22, Figure 9B). In addition, we transfected HEK-DAT cells with the Ubc9-CS inactive mutant. As shown in Supplementary Figure S6, the inactive Ubc9-CS reduced the level of DAT at the cell surface when analyzed by biotinylation (surface DAT: control = 100% ± 0.3; Ubc9-CS = 88% ± 3; Supplementary Figure S6). We also tested whether the enhanced DAT level in the plasma membrane induced by Ubc9 was correlated with an increased dopamine uptake capability. We measured the uptake capacity of DAT using [^3^H]-labeled dopamine in HEK-DAT expressing GFP or Ubc9-GFP (Figure 9C right). The kinetic analysis showed that the V_max_ was significantly increased in the Ubc9 group (V_max_ in GFP = 45.3 ± 1.4 pmol/min/well; V_max_ in Ubc9-GFP = 56.9 ± 2 pmol/min/well), while the K_m_ value was not significantly affected (K_m_ of GFP = 4.4 ± 0.5 μM; K_m_ of Ubc9-GFP = 5.6 ± 0.7 μM). In Figure 9C left, the analyses of five independent experiments showed that the relative uptake of dopamine was increased in cells overexpressing Ubc9-GFP (116% ± 1.3), relative to the GFP group (100% ± 1.5). These results support the notion that DAT steady-state level increase by Ubc9-GFP enhances the number of functional transporters in the plasma membrane. Since DAT is an electrogenic transporter, we next tested whether the increased cell surface level of DAT was associated with a rise in the electrogenic DAT activity, uncoupled to dopamine transport. We transfected HEK-DAT cells with Ubc9-EGFP or empty vector and tested whether Ubc9-GFP modulated DAT-mediated currents (Figure 9D). The families of outward currents were evoked by 200 ms voltage steps ranging from −120 to −40 mV in 20 mV increments from a holding potential of −40 mV in the control bath solution. The dopamine reuptake inhibitor nomifensine was applied to isolate DAT produced currents. The application of 10 μM nomifensine reduced outward currents (Figure 9D). Nomifensine-sensitive outward currents were obtained by subtracting the suppressed currents after the nomifensine application from the currents evoked in the control solution (Figure 9D bottom). As the current-voltage curves are shown in Figure 9E, our results demonstrate that Ubc9-GFP increased DAT uncoupled currents. Overall, our results in Figures 9 and Supplementary Figure S6 demonstrate that Ubc9 increases the functional DAT level in the plasma membrane due to the decrease of DAT degradation as shown in Figures 4, 5, 7, and 8.

## Discussion

The main goal of this study was to demonstrate that the SUMO pathway, by the action of Ubc9, increases the DAT steady-state level. The enhanced DAT level available determined a functional effect since it increased the dopamine uptake capacity because of the higher DAT expression in the plasma membrane.

Here, we analyzed the effect of DAT association with SUMO1 and the role of SUMO and the Ubc9 SUMO conjugase on DAT SUMOylation, degradation, and functional expression. In order to characterize DAT association with SUMO in a native cellular environment, we chose the immortalized rat dopaminergic N27 cell line. N27 cells have been reported as a suitable dopaminergic model in previous studies since they express TH, DAT, neuronal markers, and synthesize dopamine (Adams et al., 1996; Clarkson et al., 1998; Lazzara et al., 2015). We demonstrated that DAT is endogenously expressed in N27 cells, which is supported by means of different methods: (1) DAT was specifically immunoblotted by the long-established anti-DAT MAB (rat) antibody and the anti-DAT EL2 (rabbit) antibodies (Figures 1A,C); (2) endogenous DAT was immunoprecipitated by the EL2 DAT antibody and specifically detected by the MAB DAT antibody (Figures 4C, 5B); (3) endogenous DAT was detected by confocal microscopy in fixed cells by both EL2 (Figure 1) and MAB (data not shown) antibodies; (4) DAT mRNA in N27 cells was detected and quantified by RT-PCR (Supplementary Figure S3); (5) in cell surface biotinylation studies, endogenous DAT was present in the plasma membrane of N27 cells (Figures 1C, 8A); and (6) endogenous DAT was functional and enabled MPP^+^ uptake (Figure 1D). The significant contribution of other monoamine transporters such as NET or SERT was ruled out in the MPP^+^ uptake by using specific inhibitors (Figure 1D and Table 1). Therefore, our studies indicate that endogenous DAT is actively expressed in dopaminergic N27 cells.

We demonstrated the physical association of SUMO1 and DAT by immunoprecipitation and FRET (Figure 2). FRET is produced between two fluorophores only when they are less than 10 nm apart with a precise orientation. The spectral FRET method allowed us to visualize the full fluorescence spectrum and the corresponding energy transfer from the photobleached DAT-YFP acceptor to the SUMO1-CFP donor (Figures 2B,C). The inclusion of negative controls is essential to verify that two fluorophores produce significant FRET. Here we incorporated numerous negative and positive FRET controls, demonstrating that FRET efficiency between DAT-YFP and SUMO1-CFP was significantly positive (Figures 2C,D, and Table 2). We suggest that the association of SUMO1 and DAT might take place through the interaction of SUMO1 with a potential SUMO interactive motif (SIM) present in the DAT structure (Flotho and Melchior, 2013; Henley et al., 2014). The close association between DAT and SUMO1 prompted us to test whether SUMO1 was conjugated to DAT.

SUMO1 is an ubiquitin-like protein and has the capacity to modify proteins at one or more lysine residues as a single- or a poly-SUMO1 chain (Krumova et al., 2011; Tatham et al., 2011; Flotho and Melchior, 2013; Henley et al., 2014; Rott et al., 2017). The overexpression of DAT+SUMO1 in HEK cells allowed us to detect a strong SUMO1-DAT signal. In our studies, DAT modification by SUMO1 was detected as noticeable bands in addition to a less prominent smear background. The SUMO1-DAT pattern can be interpreted as the modification of DAT by SUMO1 on more than one residue (Miranda et al., 2005, 2007; Krumova et al., 2011; Tatham et al., 2011; Rott et al., 2017). Moreover, we were able to identify DAT SUMOylation by SUMO1 in the mouse striatum, suggesting that DAT may be endogenously SUMOylated in the brain (Supplementary Figure S1). Our experimental design allowed us to change the SUMO levels and determine how DAT degradation was correspondingly up- or down-regulated by Ubc9 level manipulations (Figures 3–5). Remarkably, SUMO overexpression and DAT SUMOylation enhanced the DAT steady-state level. This effect was observed in cells overexpressing either SUMO1 or SUMO2 (Figure 3). In addition, the overexpression of the Ubc9 SUMO-conjugase, that conjugates both SUMO1 and SUMO2, also resulted in a significant increase in DAT SUMOylation and DAT steady-state level (Figure 4). By the same token, the overexpression of the dominant negative Ubc9-CS significantly reduced DAT steady-state level and DAT expression in the plasma membrane (Figures 4A and Supplementary Figure S6). The Ubc9 knock-down by RNAi substantially reduced DAT SUMOylation and enhanced DAT degradation (Figure 5). Moreover, the level of DAT in the plasma membrane was also diminished by the Ubc9 inhibitor, 2-D08 (Figure 9A). These results clearly demonstrate that SUMOylation is involved in regulating DAT turnover.

However, it is important to point out that at this stage, our data cannot distinguish whether the increase on the DAT steady-state level is the result of direct SUMO1 conjugation to DAT or the SUMO association with DAT through a SIM in the DAT structure (Flotho and Melchior, 2013; Henley et al., 2014). The hDAT sequence of 620 aminoacids (aa) presents at least three predicted SIM: SIM1 = 31–35aa, SIM2 = 246–250aa, and SIM3 = 535–539aa. Importantly, SIM1 lies at the DAT N-terminus (_31_LILVK_35_), where lysine-35 (K35) is a target for ubiquitination (Miranda et al., 2005, 2007). Therefore, SUMO without being conjugated to DAT might associate with DAT by SIM1, impairing DAT ubiquitination and further degradation.

The predicted DAT lysines modified by SUMO in consensus and non-consensus sites, mostly reside at the N-terminus of DAT: K3, K5, K27, K19, K35, K65, and K66; with other predicted sites scattered along the DAT sequence (Flotho and Melchior, 2013; Henley et al., 2014). The DAT residues for ubiquitin conjugation is a lysine cluster composed of K19, K27, and K35 (Miranda et al., 2005, 2007). Mutagenesis of these three residues is sufficient for inhibition of PKC-dependent DAT ubiquitination and endocytosis, increasing DAT level at the plasma membrane in heterologous systems (Miranda et al., 2005, 2007). These published data clearly correspond with our results, where SUMO may outcompete ubiquitin conjugation of putative lysines at the N-terminus of DAT. The assumed competition of SUMO against ubiquitin would reduce DAT ubiquitination, endocytosis, and PKC-induced degradation as shown in Figures 6, 8.

Accordingly, we demonstrated that SUMO1 overexpression decreased DAT ubiquitination (Figure 6). Since DAT modification by ubiquitin targets DAT for lysosomal degradation, this opens the possibility of a combined regulation of DAT degradation by a balance of SUMO and ubiquitin modifications on DAT. For example, SUMO/ubiquitin modifications reciprocally regulate the degradation of proteins including α-synuclein and Huntingtin. SUMOylation counteracts α-synuclein ubiquitination produced by ubiquitin ligases such as Nedd4 (Rott et al., 2017; Rousseaux et al., 2018). Moreover, SUMO conjugation of α-synuclein may promote its accumulation and aggregation into inclusions. Importantly, in nigral neurons, aggregated α-synuclein in Lewy bodies contains SUMO1 (Kim et al., 2011). On the other hand, Huntingtin accumulation is also regulated by reciprocal modifications exerted by SUMO and ubiquitin. Huntingtin SUMOylation impairs its degradation by the proteasome, increasing Huntingtin aggregation and toxicity (Steffan et al., 2004; O’Rourke et al., 2013). Yet, another example is the IkB-α transcriptional inhibitor which is modified by SUMO, impairing its degradation (Desterro et al., 1998). As a consequence, SUMO overexpression stabilizes IkB-α and impairs the activation of a critical transcription factor (Desterro et al., 1998). Future studies will lead to the characterization of DAT degradation regulated by SUMO/ubiquitin reciprocal modifications and the putative DAT residues conjugated by SUMO that might correspond to the characterized ubiquitin-targeted lysines in the DAT N-terminus (Miranda et al., 2005, 2007).

Since the Ubc9 activity has been demonstrated to be a factor for protein quality control, we assessed whether Ubc9 affected DAT proteasomal degradation (Hay, 2005; Yan et al., 2005; Tatham et al., 2008, 2011; Ahner et al., 2013; Atkin and Paulson, 2014; Gupta et al., 2014). The proteasomal degradation of membrane proteins is mostly related to endoplasmic reticulum-associated degradation (ERAD), which is based on protein retention in the ER, followed by poly-ubiquitination and degradation by the proteasome (Hay, 2005; Yan et al., 2005; Atkin and Paulson, 2014; Gupta et al., 2014). DAT degradation by ERAD is physiologically relevant since the amount of DAT leaving the ER would influence the amount of DAT localized in the plasma membrane, as it has been demonstrated for other membrane proteins (Hay, 2005; Yan et al., 2005; Ahner et al., 2013; Atkin and Paulson, 2014; Gupta et al., 2014; Ciechanover and Kwon, 2015). Human DAT mutations associated with DAT misfolding and degradation have been recently described as causes for the hereditary DTDS (Kurian et al., 2009, 2011; Ng et al., 2014; Beerepoot et al., 2016; Kasture et al., 2016). The DAT mutations linked to DTDS, impair the DAT trafficking to the cell surface because of ER retention and degradation, possibly by ERAD. It would be important in future research to determine the intricate mechanisms of DTDS-causing mutations and whether this is related to DAT SUMOylation. Our data suggest that the proteasome is clearly involved in regulating DAT degradation. Proteasomal inhibition by MG132 significantly decreased DAT degradation in both GFP and Ubc9-GFP N27 cells. Interestingly, MG132 in combination with Ubc9-GFP almost completely blocked DAT degradation (Figure 7). Although our statistical analysis supports a possible additive influence of Ubc9 on the MG132 treatment, it appears that both effects on DAT degradation are independent of each other since no significant interaction was confirmed. Therefore, additional studies will be needed to determine whether SUMOylation may regulate DAT degradation through the proteasome pathway (Hay, 2005; Tatham et al., 2008, 2011; Ahner et al., 2013; Gupta et al., 2014).

Our results suggest that SUMOylation enhances the DAT steady-state level by impairing DAT degradation through lysosomes. A number of reports have demonstrated that DAT internalization and targeting to lysosomes is strongly influenced by the cellular milieu where it occurs (Daniels and Amara, 1999; Miranda et al., 2005, 2007; Eriksen et al., 2009; Gabriel et al., 2013; Block et al., 2015; Wu et al., 2015). Recent studies have also shown that PKC activation by PMA induced DAT degradation in the absence of endocytosis (Foster and Vaughan, 2011). Accordingly, our results support that DAT lysosomal degradation induced by PKC is prevented by Ubc9 overexpression (Figure 8A). In addition, studies performed in dopaminergic neurons suggest that DAT internalization is not subject to regulation by PKC, in contrast to the effect identified in heterologous systems (Eriksen et al., 2009; Gabriel et al., 2013; Block et al., 2015). In conjunction with those reports, we suggest that the resistance of DAT to PKC-mediated lysosomal degradation in neurons may be explained by their relatively high expression of Ubc9-mRNA when compared to non-neural cells as demonstrated by RT-PCR mRNA quantification (Dustrude et al., 2013).

We also demonstrated that Ubc9 overexpression induced an apparent DAT resistance to PKC triggered internalization (Figure 8B). It has been shown that endocytosis can be regulated by direct SUMOylation in a number of membrane proteins, either increasing or decreasing internalization (Martin et al., 2007; Kruse et al., 2009; Dustrude et al., 2013; Sun et al., 2014). For example, PKC-α has been shown to be inhibited by SUMOylation, impairing the internalization of glycine receptors in neurons (Sun et al., 2014). In addition, as the inhibition of mitogen-activated protein kinase (MAPK) reduces the dopamine uptake by DAT, it would be interesting to assess whether MAPK exerts its regulation on DAT SUMOylation levels (Morón et al., 2003; Kim et al., 2011). However, the identified Ubc9 inhibitory effect on DAT internalization has to be interpreted carefully (Figure 8B). Since Ubc9 also reduces DAT degradation increasing its steady-state level (Figures 4, 5), DAT anterograde trafficking may be enhanced, partially compensating the DAT internalization effect of PMA (Yan et al., 2005; Zahniser and Sorkin, 2009).

Since our results have been mostly collected in a cellular system, it would be interesting to further investigate the addressed mechanisms *in vivo*. Based on our study, the SUMO/Ubc9 overexpression and the consequent DAT SUMOylation described here is a new mechanism on how DAT is functionally regulated in the plasma membrane. Therefore, the SUMO pathway appears as a regulatory mechanism and a potential therapeutic target for regulating DAT function, dopamine clearance at the presynaptic terminal, and dopamine signaling in the pathophysiology of autism spectrum disorders, ADHD, and DTDS.

In summary, we demonstrate that: (1) N27 dopaminergic cells express functional DAT, which is physically associated or conjugated to SUMO1; (2) SUMO1, SUMO2, and Ubc9 overexpression enhance DAT steady-state level; (3) Ubc9/SUMO1 overexpression increases DAT SUMOylation, decreasing DAT degradation; (4) Ubc9 knock-down by RNAi reduces DAT SUMOylation and increases DAT degradation; (5) SUMO1 overexpression decreases DAT ubiquitination level; (6) the effect of Ubc9/SUMO1 on enhancing DAT steady-state level may be derived from a reduction in DAT lysosomal degradation; (7) in the plasma membrane, Ubc9 may impair PKC-mediated DAT internalization; and (8) the enhanced DAT steady-state level determines an increased functional activity of DAT in the plasma membrane.

## Author Contributions

EC, Y-HK, JG-O, ML, JC, and MM contributed to experimental designs, data acquisition, analysis, and interpretation. EJ and JV contributed to data acquisition and analysis. EC and GT contributed to project conceptualization. Y-HK contributed to critical revisions and final approval and had overall responsibility for the article. EC and Y-HK wrote the article. All authors read and approved the final version of the manuscript.

## Conflict of Interest Statement

The authors declare that the research was conducted in the absence of any commercial or financial relationships that could be construed as a potential conflict of interest.

## Supplementary Material

FIGURE S1 SUMO1 is conjugated to DAT in the mouse striatum. Mouse striatum extracts were immunoprecipitated with rabbit anti-SUMO1, rabbit anti-DAT, or rabbit IgG antibodies and blotted with anti-DAT (MAB). The SUMO-DAT is indicated with a bracket and non-specific bands are indicated with stars. Striatum DAT lysate inputs for each individual assay are shown. Right panel shows 10% of total lysate for the SUMO1 present in the mouse striatum, detected with rabbit anti-SUMO1. The free SUMO1 is indicated with a single star and SUMO1 conjugated species are indicated with a bracket (right). The immunoprecipitation assay was repeated more than three times.

FIGURE S2 Ubc9-GFP overexpression increases DAT protein levels by preventing its degradation in N27 cells. A representative image portraying DAT levels in both GFP and Ubc9-GFP N27 cell lines in one single membrane. Cells were incubated with cycloheximide and DAT was chased at *T* = 0 (100% initial protein) and 24 h (*T* = 24 h). DAT level was detected with rabbit anti-DAT (EL2) antibody and β-actin was used as an equal loading control.

FIGURE S3 Ubc9-GFP does not impact DAT transcription. A quantitative real-time PCR (qRT-PCR) was performed to determine the level of DAT mRNA, with β-actin as a housekeeping gene. The mRNA ratio of DAT/ β-actin was determined by fluorescence of SYBR-green (three independent experiments). ns, not significant.

FIGURE S4 Both SUMO1 and SUMO2 overexpression reduce DAT ubiquitination. A representative image of immunoprecipitations performed using HEK cell lysates transfected with both DAT and ubiquitin to improve the recovery of DAT-ubiquitin. DAT-ubiquitin was immunoprecipitated by mouse anti-ubiquitin antibody in cells transfected with or without SUMO1-HA or SUMO2-HA. Recovered DAT-ubiquitin was detected with anti-DAT (MAB) antibody. Inputs for DAT, free ubiquitin, and β-actin are displayed. Free ubiquitin was detected with mouse anti-ubiquitin antibody. β-actin as a loading control is shown at the bottom. There is a decrease on the recovered DAT-ubiquitin level when SUMO1-HA or SUMO2-HA is overexpressed. The figure is a representative image of three independent experiments.

FIGURE S5 Ubc9 prevents PMA-induced DAT degradation in N27 cells. A representative image showing DAT in a cycloheximide chase for 2 h, from both GFP and Ubc9-GFP cell lines, in one single membrane. In the cycloheximide chase, incubation with or without 2 μM PMA had a differential effect on DAT depending on whether Ubc9-GFP was overexpressed or not. Ubc9-GFP overexpression prevents the PMA-induced DAT degradation.

FIGURE S6 Surface biotinylated DAT level was significantly reduced with Ubc9-CS overexpression. HEK-DAT cells were transfected with either the mutant Ubc9 C26S or empty vector. Cell surface biotinylation was performed with non-permeable sulfo-NHS-biotin. Surface biotinylated DAT was immunoblotted with anti-DAT (MAB) antibody. Total inputs for DAT are shown. Data represent mean ± SE and statistical significance from control (**p* < 0.05) was determined by a two-sided, Student’s *t*-test. Values were normalized as average ± SE (three independent experiments).

## Abbreviations

PMA: phorbol 12-myristate 13-acetate
PKC: protein kinase C
EGFP: enhanced green fluorescent protein
HEK: human embryonic kidney cells
PD: pixel density
DAT: dopamine transporter
Chloroq: chloroquine
MAB: rat monoclonal anti-dopamine transporter
EL2: rabbit polyclonal anti-dopamine transporter
MPP^+^: methyl-4-phenylpyridinium.
