# Supplementary figures and images for: The SUMO-Conjugase Ubc9 Prevents the Degradation of the Dopamine Transporter, Enhancing Its Cell Surface Level and Dopamine Uptake
Source: Front Cell Neurosci. 2019 Feb 8;13:35. doi: 10.3389/fncel.2019.00035 (PMC6386010; doi:10.3389/fncel.2019.00035)

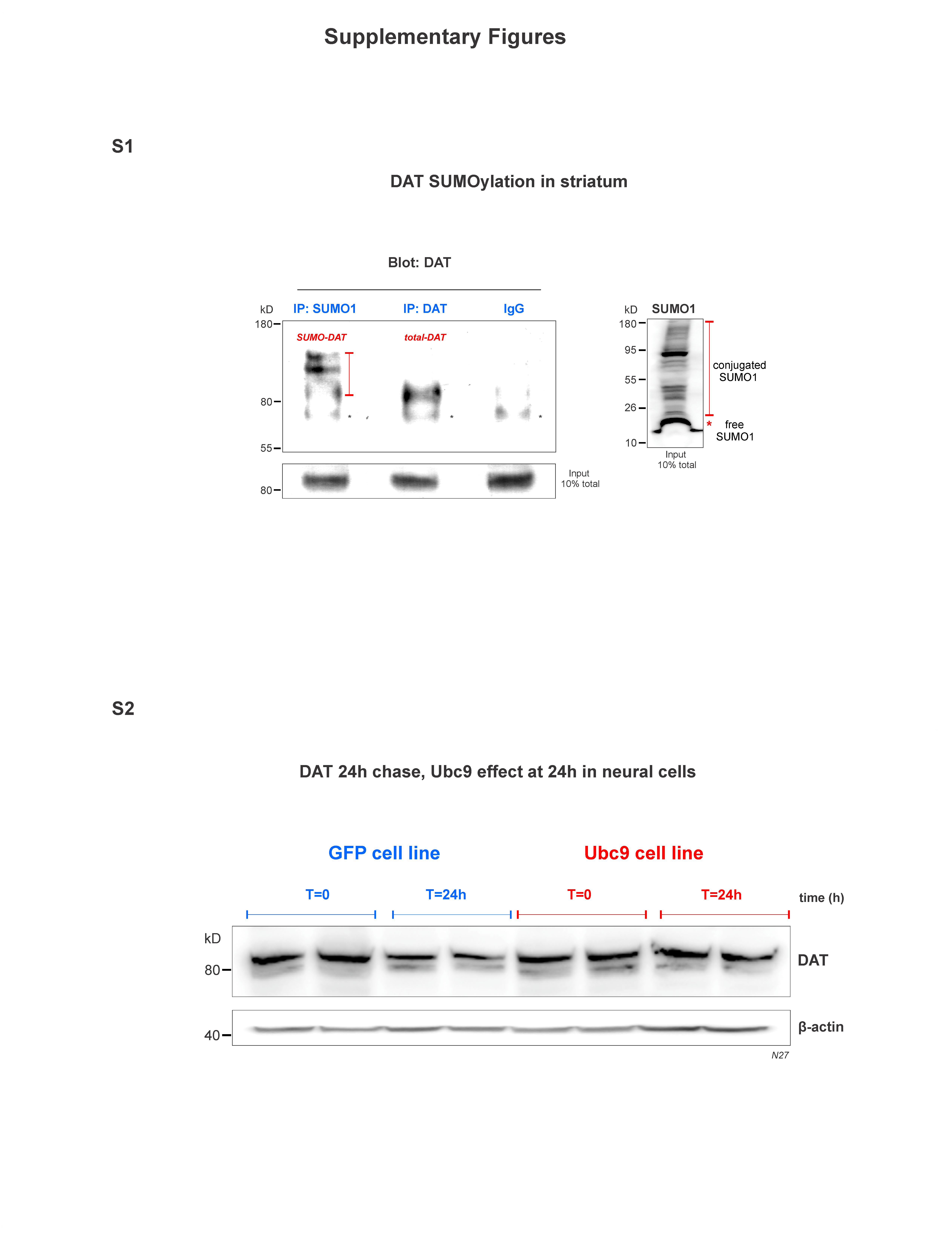

Supplement: FIGURE S1 — SUMO1 is conjugated to DAT in the mouse striatum. Mouse striatum extracts were immunoprecipitated with rabbit anti-SUMO1, rabbit anti-DAT, or rabbit IgG antibodies and blotted with anti-DAT (MAB). The SUMO-DAT is indicated with a bracket and non-specific bands are indicated with stars. Striatum DAT lysate inputs for each individual assay are shown. Right panel shows 10% of total lysate for the SUMO1 present in the mouse striatum, detected with rabbit anti-SUMO1. The free SUMO1 is indicated with a single star and SUMO1 conjugated species are indicated with a bracket (right). The immunoprecipitation assay was repeated more than three times. [file Image_1.TIF]

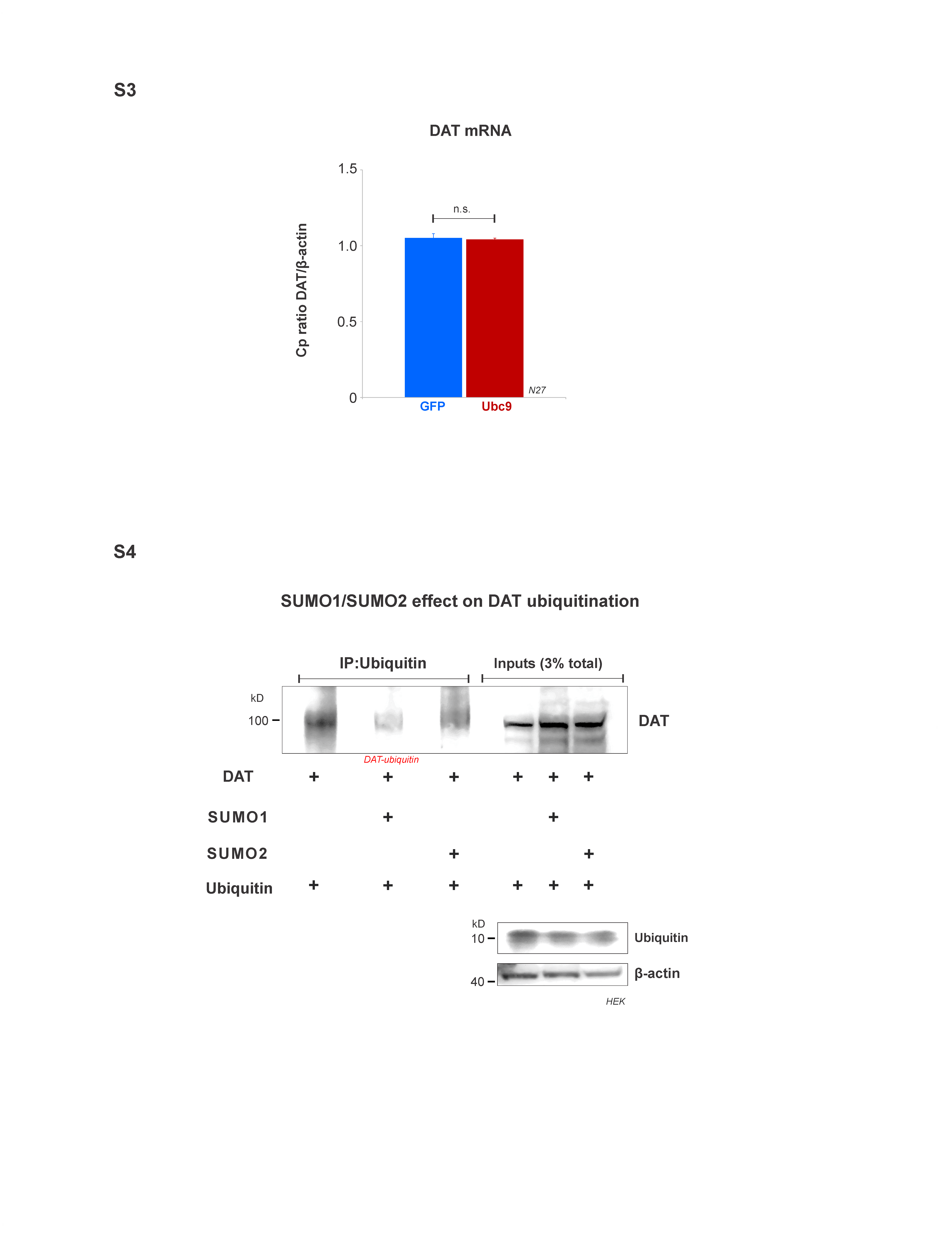

Supplement: FIGURE S3 — Ubc9-GFP does not impact DAT transcription. A quantitative real-time PCR (qRT-PCR) was performed to determine the level of DAT mRNA, with β-actin as a housekeeping gene. The mRNA ratio of DAT/ β-actin was determined by fluorescence of SYBR-green (three independent experiments). ns, not significant. [file Image_2.TIF]

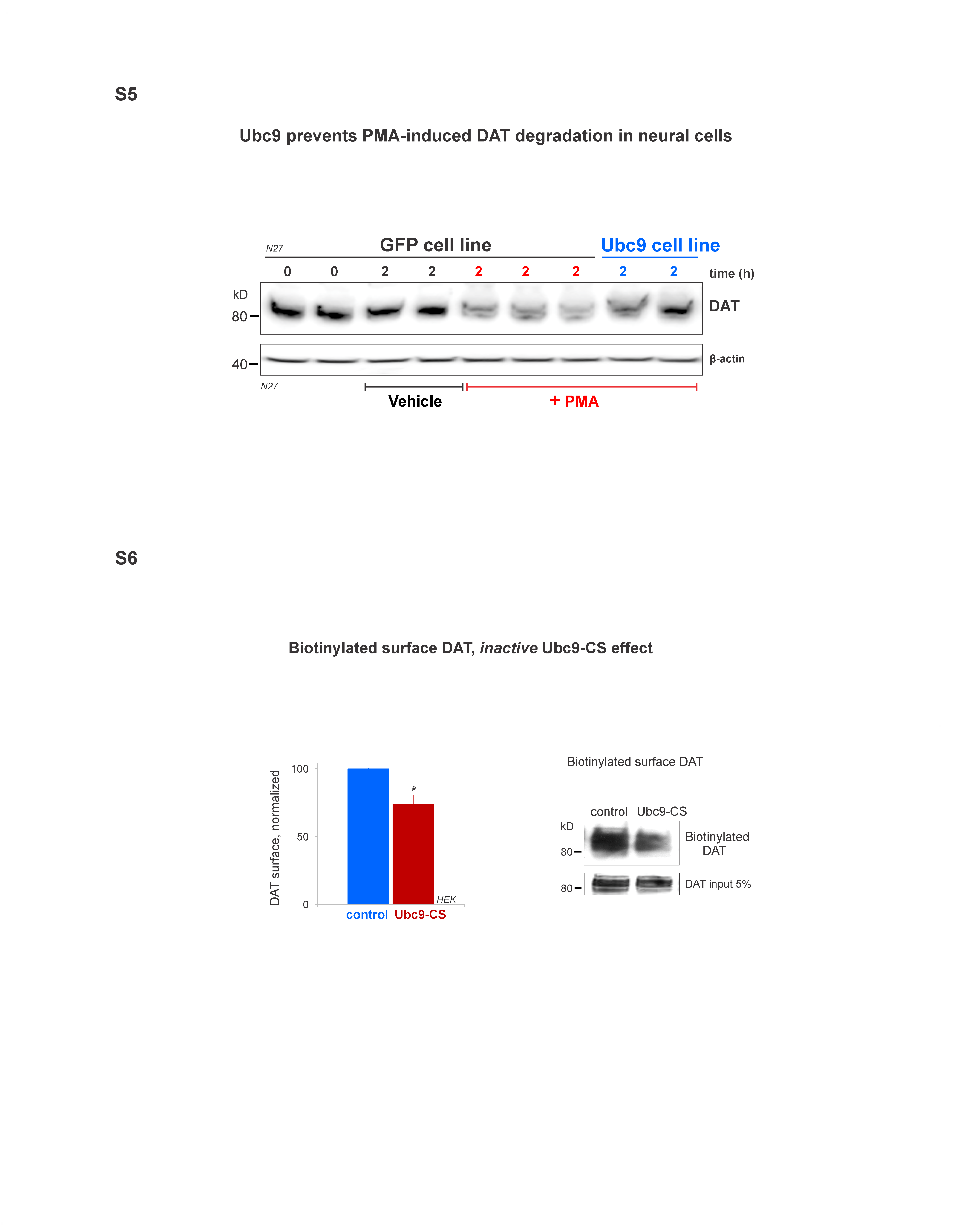

Supplement: FIGURE S5 — Ubc9 prevents PMA-induced DAT degradation in N27 cells. A representative image showing DAT in a cycloheximide chase for 2 h, from both GFP and Ubc9-GFP cell lines, in one single membrane. In the cycloheximide chase, incubation with or without 2 μM PMA had a differential effect on DAT depending on whether Ubc9-GFP was overexpressed or not. Ubc9-GFP overexpression prevents the PMA-induced DAT degradation. [file Image_3.TIF]
